# The use of routine health facility data for micro-stratification of malaria risk in mainland Tanzania

**DOI:** 10.1186/s12936-022-04364-7

**Published:** 2022-11-18

**Authors:** Sumaiyya G. Thawer, Monica Golumbeanu, Khalifa Munisi, Sijenunu Aaron, Frank Chacky, Samwel Lazaro, Ally Mohamed, Noela Kisoka, Christian Lengeler, Fabrizio Molteni, Amanda Ross, Robert W. Snow, Emilie Pothin

**Affiliations:** 1 Swiss Tropical and Public Health Institute, Allschwil, Switzerland; 2 University of Basel, Basel, Switzerland; 3 Ministry of Health, Dodoma, Tanzania; 4 National Malaria Control Programme, Dodoma, Tanzania; 5 Population Health Unit, Kenya Medical Research Institute-Wellcome Trust Research Programme, Nairobi, Kenya; 6 Centre for Tropical Medicine and Global Health, Nuffield Department of Clinical Medicine, University of Oxford, Oxford, UK; 7 Clinton Health Access Initiative, New York, USA

**Keywords:** Malaria, Micro-stratification, Routine data, Tanzania

## Abstract

**Background:**

Current efforts to estimate the spatially diverse malaria burden in malaria-endemic countries largely involve the use of epidemiological modelling methods for describing temporal and spatial heterogeneity using sparse interpolated prevalence data from periodic cross-sectional surveys. However, more malaria-endemic countries are beginning to consider local routine data for this purpose. Nevertheless, routine information from health facilities (HFs) remains widely under-utilized despite improved data quality, including increased access to diagnostic testing and the adoption of the electronic District Health Information System (DHIS2). This paper describes the process undertaken in mainland Tanzania using routine data to develop a high-resolution, micro-stratification risk map to guide future malaria control efforts.

**Methods:**

Combinations of various routine malariometric indicators collected from 7098 HFs were assembled across 3065 wards of mainland Tanzania for the period 2017–2019. The reported council-level prevalence classification in school children aged 5–16 years (*Pf*PR_5–16_) was used as a benchmark to define four malaria risk groups. These groups were subsequently used to derive cut-offs for the routine indicators by minimizing misclassifications and maximizing overall agreement. The derived-cutoffs were converted into numbered scores and summed across the three indicators to allocate wards into their overall risk stratum.

**Results:**

Of 3065 wards, 353 were assigned to the very low strata (10.5% of the total ward population), 717 to the low strata (28.6% of the population), 525 to the moderate strata (16.2% of the population), and 1470 to the high strata (39.8% of the population). The resulting micro-stratification revealed malaria risk heterogeneity within 80 councils and identified wards that would benefit from community-level focal interventions, such as community-case management, indoor residual spraying and larviciding.

**Conclusion:**

The micro-stratification approach employed is simple and pragmatic, with potential to be easily adopted by the malaria programme in Tanzania. It makes use of available routine data that are rich in spatial resolution and that can be readily accessed allowing for a stratification of malaria risk below the council level. Such a framework is optimal for supporting evidence-based, decentralized malaria control planning, thereby improving the effectiveness and allocation efficiency of malaria control interventions.

**Supplementary Information:**

The online version contains supplementary material available at 10.1186/s12936-022-04364-7.

## Background

The future of malaria control and elimination depends on characterizing the level of disease risk in time and space, which should be constantly reviewed to guide optimal, tailored malaria control strategies specific to sub-national settings [1, 2]. Traditionally, malaria parasite prevalence data among community residents, collected through periodic cross-sectional surveys, has been used to characterize malaria ecologies sub-nationally [3–7]. Over the last 20 years, increasingly complex, model-based, geo-statistical approaches [8, 9] have been applied to assembled community parasite prevalence data to provide interpolated data for high-resolution malaria risk maps [10–13]. These approaches have been commonly used at national levels in providing national malaria control programmes (NMCPs) with baseline information on infection risk for various decision-making and planning purposes [14–24].

However, community parasite prevalence data are collected nationally only periodically every 2–3 years and household sampling strategies lack power for small area estimation. Data are therefore sparse in time and space, and unable to describe the malaria situation continuously and at fine spatial resolutions with precision. A more ubiquitous source of information derives from routine health service data, collected continuously at most populated locations. These data provide a rich source of malariometric indicators in different population age and risk groups. Outside of countries aiming for malaria elimination, where individual case detection is a fundamental requirement, most stable endemic countries have not fully exploited routine data to its full potential. This was largely due to issues with the quality of the data and their completeness [25–27]. In recent years, these concerns have been tackled across sub-Saharan Africa (SSA) due to various factors such as the launch of the revitalized WHO policy of test-treat-track [28] that has increased testing rates, the transition towards the electronic district health information system (DHIS2) that has improved health reporting rates [29] and the implementation of continuous data quality assessments [30]. Consequently, routine data are now increasingly recommended and used for national stratification of malaria risk and decision-making [31–37].

Most national stratifications of malaria risk have considered one or two administrative levels (province, region, district, council) and are called ‘macro-stratification’ here. These often correspond to the federal planning of control and resource allocation levels [32]. However, marked epidemiological risk heterogeneity has been seen at these levels, and a lower level stratification has been proposed: micro-stratification [38–40]. Malaria transmission is spatially heterogeneous in its distribution at every scale, driven by local ecologies, climate and population settlement [41–45]. With an increasing empowerment of decentralized health sector governance and recognizing the small area variations in malaria risk, there is a need to improve our abilities to develop more detailed data platforms and risk analyses [46]. Such a more granular stratification of malaria risk will allow for better spatially targeted malaria control responses and hence improve effectiveness and allocation efficiency.

Complex modelling approaches of parasite prevalence are often challenged by limited national capacity and ownership issues [14, 47, 48]. As NMCPs are gaining more analytic capacity and confidence in using routine DHIS2 data, including the local development of embedded malaria dashboards, quality checks and monthly/quarterly reports, this situation is changing [49–51]. Statistical modelling of routine health data, spatially and temporally, in low-income countries is in its nascent stages and largely driven by partners outside of malaria-endemic countries. Data analytics for NMCPs must be transparent and straightforward, as well as guided by principles of completeness, coverage and inter-operability between various malaria indicators.

This work builds upon previous effort started as a collaborative exercise with the Tanzanian NMCP [37] to improve the use of routine malaria indicators from DHIS2, and propose a novel, pragmatic and data-rich method for implementing malaria risk micro-stratification below council levels.

## Methods

### Context

In 2017, during a mid-term review of the national malaria strategic plan (NMSP) [52] followed by a malaria expert consultative meeting [53], it was recognized that in order to sustain Tanzania’s reductions in malaria burden, a more geographic-tailored package of interventions was needed. This led to a country-managed, data-driven approach to develop a macro-stratification malaria risk map at the second level of administrative unit, across 184 councils [37, 54, 55]. Each council was assigned to one of four risk strata: very low, low, moderate, and high. An assembly of survey data from available prevalence surveys, together with routine data was used to define the four risk categories by means of expert-informed empirical ranges of malaria prevalence in school children (*Pf*PR_5–16yrs_). Routine data included fever test positivity rates (TPR), annual parasite incidence (API) and antenatal attendee test positivity rates (ANC TPR). Based on this novel approach to using multiple data sources [36], a revised NMSP was issued in 2018 [56]. Additional work and consultative processes, as well as intervention mix optimization in each risk strata using stochastic modelling [54, 55] led to the development of the NMSP for 2021–2025 [57]. As per NMSP recommendation, the stratification exercise should be renewed every 3 years, to account for the changing epidemiology of the disease. To extend analytics and support the decentralized health system in Tanzania, the NMSP recommended approaches are repeated for risk stratification at ward levels to account for intra-council heterogeneity.

### Administrative boundaries and populations at risk in mainland Tanzania

Mainland Tanzania is organized into multiple administrative levels. The country has 26 administrative regions, divided into 184 councils. Councils serve as the key operational unit for central government resource allocation and planning disease prevention and management activities, with own budgeting abilities. Councils are further divided into wards, which serve as the lower levels of administrative resource units and disease reporting. A total of 3311 wards have been defined according to the 2012 national census for mainland Tanzania. Out of these, 2427 are rural, 370 are mixed and 514 are urban (Additional file 1: Fig. S1). The number of wards per council range from two to 43 wards depending on the size of the council, and these allow for a much more granular risk definition, especially in areas with marked altitudinal variation. Each ward, depending on its size, includes between one to 18 health facilities (HFs) that serve the surrounding village populations. Unfortunately, the precise HF catchment population remains largely undefined, and aggregated population units for each ward was therefore used for the micro-stratification process. The population for each ward was obtained from the publicly available 2012 population and housing census in Tanzania conducted by the National Bureau of Statistics [58]. Annual growth rates at the council level (computed from the average annual continuous growth rate formula) were applied to the ward population data to project each ward population to the period 2017–2019. This allowed the compute of the denominators for API calculations, and to quantify populations residing in the ward malaria risk classifications.

### Routine health facility data processing

Since 2009, the health management and information system (HMIS) of Tanzania has seen an evolution from a paper-based system to the electronic DHIS2 system. DHIS2 is an open source, web-based software platform for reporting, analysis and dissemination of health data. It captures information from both the private (26%) and public (74%) HFs and can be accessed by officials working in the health sector, through registered credentials. The work presented here utilized key malaria data extracted from the HMIS/DHIS2: the total number of *falciparum* malaria laboratory-confirmed cases, total number of malaria rapid diagnostic tests (RDTs) performed, and total number of confirmed cases and RDT tests performed in pregnant women attending antenatal care (ANC) during their first visits. These data were used to compute three malaria indicators: API, RDT TPR and ANC TPR (details presented in Table 1). Since the majority of reporting HFs [N = 7878 (99%)] providing laboratory services in Tanzania use RDT as the main diagnostic test (88% of total tests performed), and routine microscopy is prone to quality issues [59], only RDT test results were considered for the micro-stratification analysis.

**Table 1:** Indicators used for malaria risk micro-stratification

Source	Indicator	Numerator	Denominator	Period^a^	Age	Level
HMIS/DHIS2	Laboratory					
	Fever test positivity rate (RDT TPR)	No. positive *Pf*-pan RDT	No. *Pf-*Pan RDT tests performed	2017–2019	All ages	Council and Ward
	Annual parasite incidence (API)	No. positive *Pf*-pan RDT	Per 1000 population^b^			
	Antenatal clinic					
	Test positivity rate (ANC TPR)	No. positive *Pf*-pan RDT	No. *Pf*-Pan RDT tests performed in pregnant women at first visit	2017–2019	Reproductive age	Council and ward
SMPS	Parasite prevalence	No. positive *Pf*-pan RDT	No. *Pf*-Pan RDT tests performed in school children	2017, 2019	5–16 years	Council

*HMIS* Health Management Information System, *DHIS2* District Health Information System 2, *RDT* malaria Rapid Diagnostic Test, *Pf* *Plasmodium falciparum*, *SMPS* School Malaria Parasitaemia Survey

^a^Periods refer to 1 January to 31 December of the corresponding year

^b^Based on population estimates from the 2012 census

### Data cleaning

Routine malaria data were extracted directly from DHIS2 from a total of 7988 (94%) reporting HFs for each month for the period January 2017 to December 2019. Duplicate reports and HFs with no testing performed in any of the 36 reporting months were excluded. As the DHIS2 database is unable to distinguish zeros from missing values marking them as blank, it was assumed that missing values of otherwise complete reports were true zeros. A threshold of 50% completeness of reporting across 36 months was used and any HFs with reporting less than this were excluded from the analysis. Furthermore, HFs with more than 5 consecutive months of missing reports within a year were also excluded from the analysis. Extreme outliers, defined as monthly values that significantly deviated from the HF’s overall time series trend across the 36 months, were excluded using the R package *anomalize* [60] (Additional file 1: Text S1) and visually verified before being subsequently treated as a missing monthly report.

### Data aggregation

Geographical coordinates of the HFs were obtained from the master HF list of Tanzania [61] and linked to the DHIS2 data using the unique HF identifier code. The ward shape file was then used to allocate the HFs to their respective wards (Additional file 1: Fig. S2). Monthly data of the total malaria tests performed and those tested positive from all HFs were aggregated to provide annualized estimates per council and per ward for the reporting period (2017–2019) and subsequently used to compute the three selected routine malaria indicators: (1) RDT TPR; (2) API; and, (3) ANC TPR (definitions of these indicators are presented in Table 1). The monthly data were aggregated for the whole year in order to align with the national strategic plan development and review cycle every 3 years and provide risk estimates for the period of analysis. The council level estimates were used to derive the cut-offs for categorizing the routine indicators as per the school prevalence classifications (see details of process below) whilst ward level estimates were used for the micro-stratification. A pragmatic, conservative approach was taken to ensure that the maximum ward value from the 3 years for each indicator was used. Taking the highest of the three annual ward values to reflect the ward estimate for the period of analysis ensured that wards were rather over- than under-allocated into risk strata.

### The micro-stratification procedure

The micro-stratification risk scoring was developed in three steps: (a) suitable cut-offs were defined to allocate the three routine indicators into four risk categories, based on a pre-classification on the basis of prevalence values in school children; (b) the three selected routine indicators assigned to four malaria risk categories were converted into numbered scores; and, (c) for each ward, the total score was summed across the three corresponding malaria indicators to obtain an overall score that was used to assign each ward to a risk stratum (very low, low, moderate or high), based on scoring thresholds (see definitions below). The strategic approach undertaken was purposively designed to ensure that the approach was simple and could easily be adapted by the NMCP and health planners at council levels.

### Definition of indicator cut-offs for malaria risk categorization at the council level

In the micro-stratification process, the classification of prevalence in school children (*Pf*PR_5–16_) was used as a gold standard in guiding the selection of appropriate cut-offs for converting the three routine malaria indicators into risk categories. In mainland Tanzania, nationwide school malaria parasitaemia surveys (SMPS) targeting public primary school children have been conducted biennially since 2014 [62]. Schools were sampled based on (1) existing public primary schools in each council, and (2) expected malaria endemicity [62–64] to provide credible estimates of infection prevalence in ages 5–16 years for each of the 184 councils. Because of the quality and comprehensiveness of these data, as well as the fact that they were collected concurrently with the routine data, they served as a ‘gold’ standard for categorizing the routine indicators. Since SMPS results were available at council level, the risk categorization of the three routine indicators was also done first at council level.

The maximum prevalence in school children estimated per council across the past two surveys conducted in 2017 and 2019 was used to define stringent baseline cut-offs for each of the three routine indicators in a systematic process. Firstly, the prevalence in school children was used to define four malaria risk groups: very low (*Pf*PR_5–16_ < 1%), low (*Pf*PR_5–16_ 1 to < 5%), moderate (*Pf*PR_5–16_ 5 to < 30%), and high (*Pf*PR_5–16_ ≥ 30%) and each council was categorized into one of these four risk levels. These endemicity cut-offs were guided by WHO classifications along with consultative discussions between NMCP and malaria experts [37, 56, 65].

Secondly, in order to identify the best routine data cut-offs, a misclassification analysis was undertaken against school prevalence categories at the council level. For each routine indicator, the sensitivity, specificity, false positivity rate (FPR), and false negativity rate (FNR) were calculated per risk group for a range of cut-off values to ensure that the most robust cut-off values were selected (Additional file 1: Table S1). The selection of robust cut-offs for the routine malaria indicators was guided by a set of criteria, relevant for malaria control: (i) maximizing the specificity in the very low and low strata to reduce false positive councils in these strata; (ii) maximizing the sensitivity in moderate and high strata in order to reduce the number of false negative councils; and (iii) maximizing the overall agreement of the risk groups between school prevalence and routine indicators. These criteria ensured to minimize the misallocation of councils belonging to the higher strata to the lower strata where the largest changes in the intervention packages are seen and was termed as unacceptable (Additional file 1: Text S2). For instance, when selecting the optimal cut-off to define the very low and low risk category for the routine indicators, the criteria was based on trade-offs for minimization of FPR of councils with *Pf*PR_5–16_ > 1% and *Pf*PR_5–16_ > 5%, respectively, into the lower risk category and maximization of the overall agreement between indicators. Similarly, when selecting the optimal cut-off to define the moderate and high categories for the routine indicators, the criteria were based on trade-offs between minimization of FNR of councils with *Pf*PR_5–16_ > 30% to the lower risk category and maximization of the overall agreement between indicators.

Following the selection of suitable cut-offs for all the routine indicators at the council level, the same cut-offs were applied to the routine indicators at the ward level to categorize them into their respective risk groups at that level.

### Assignment of risk scores at the ward level

In order to combine the risk categories of the three routine indicators into a single stratum value per ward, a combined scoring approach was used for each ward. This entailed assigning numbered scores from 1 to 4 to each indicator per ward, corresponding to the respective risk categories: ‘very low’ (score 1), ‘low’ (score 2), ‘moderate’ (score 3), and ‘high’ (score 4).

### Combination of routine indicators

To obtain the overall malaria risk score per ward, the assigned indicator scores were summed across the indicators. The total score ranged from 3 to 12 and was grouped into four risk categories to form the epidemiological strata. Specifically, wards with an overall score ≤ 3 were allocated to the very low stratum, > 3 to ≤ 6 to the low stratum, > 7 to ≤ 9 to moderate stratum, and > 9 in the high stratum. Since not all wards had HFs with both ANC and laboratory services, the number of routine indicators per ward differed. As a result, the sub-division of the total score to classify the wards to the overall risk strata differed for those wards with fewer than three routine indicators (Additional file 1: Table S2).

### Quantification of malaria risk heterogeneity within councils

In order to identify the councils that had the largest variation of malaria risk within their boundaries, the proportion of wards with different ward-level risks was quantified. This heterogeneity was computed by calculating the number of wards assigned to the moderate and high transmission strata occurring within the councils with *Pf*PR_5–16_ < 5% and the number of wards assigned to the very low and low transmission strata occurring within councils with *Pf*PR_5–16_ ≥ 5%. The corresponding proportion of the total population residing in these wards was also quantified.

R Studio [66] was used for cleaning and analysis of the data downloaded from DHIS2. All maps were produced using the QGIS software version 3.4.14 [67].

## Results

### Coverage and completeness of routine HMIS/DHIS2 data

**Fig. 1:**
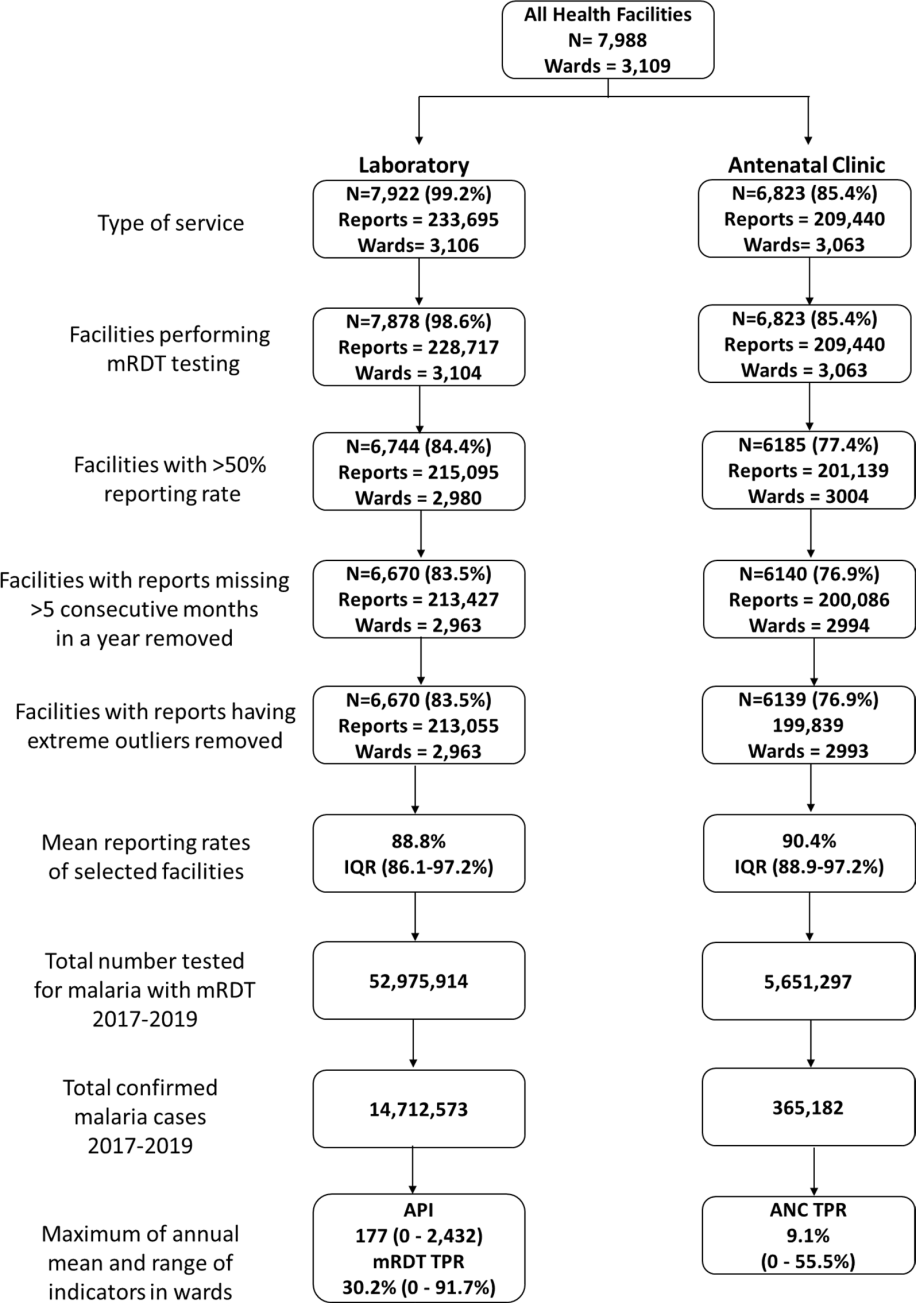
Descriptive summary of health facilities for which malaria data were utilized for micro-stratification. *ANC* antenatal clinic, *IQR* interquartile range, *API* annual parasite incidence, *TPR* test positivity rate

Figure 1 provides a descriptive summary of the HFs and indicators included in the micro- stratification.

Of the 7988 geo-coded HFs, the geo-coordinates for the majority (85%) were obtained from the master HF registry, while for 11% of HFs, the geo-coordinates did not match the indicated ward name in the master HF registry and therefore adjusted accordingly to reflect the indicated ward. A large proportion of these HFs offering malaria services belonged to the public health sector (72%), with 26% belonging to the private sector and 2% whose ownership status was not known at the time of analysis. Dispensary and clinics represented most of all the HFs (85.7%), followed by health centers (10.7%) and hospitals (3.6%) (Additional file 1: Fig. S2).

**Fig. 2:**
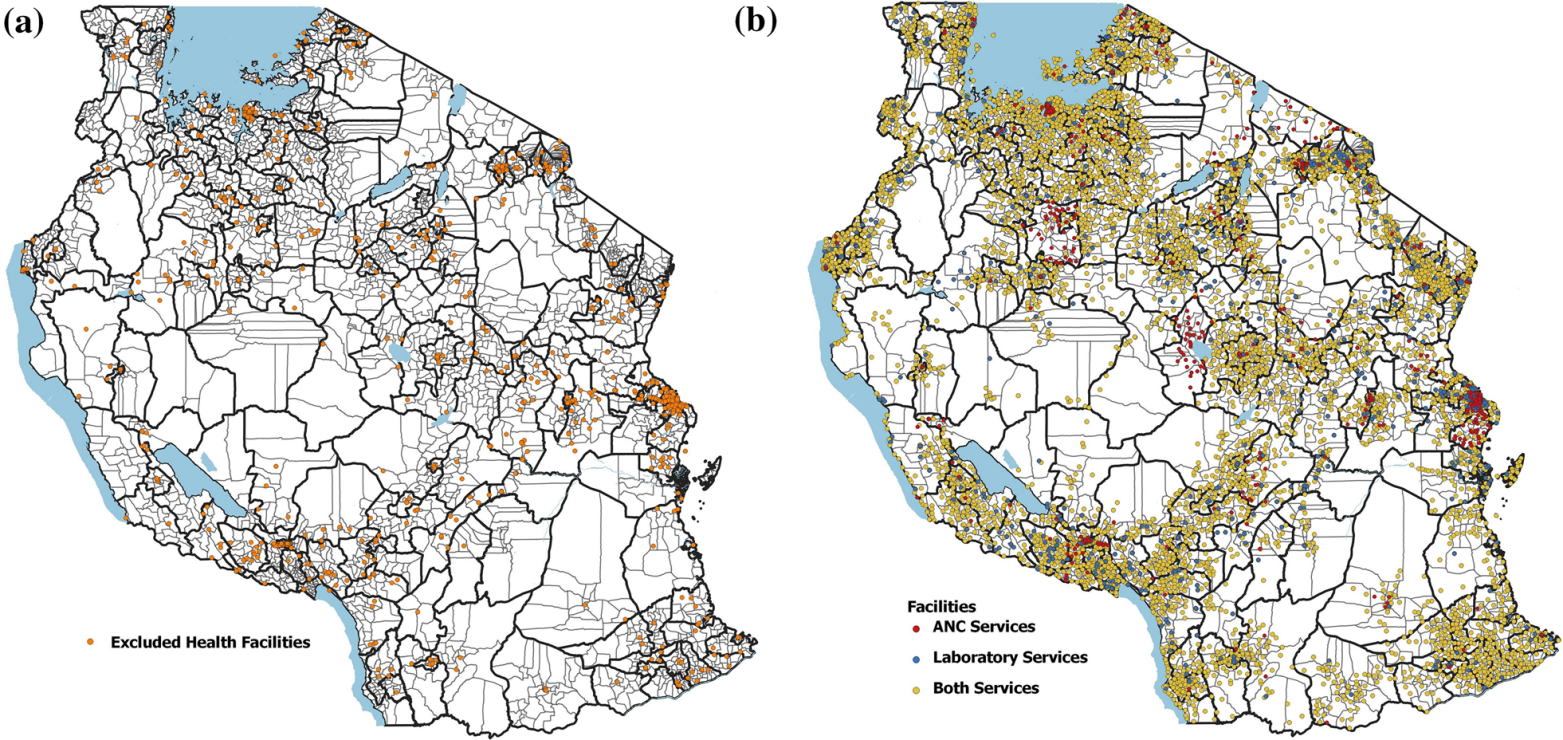
**a** Location of health facilities that were excluded (N = 890). **b** Location of health facilities by type of service that were utilized for micro-stratification (N = 7098). *ANC* antenatal clinic

Out of the total HFs, 7878 HFs (98.6%) across 3104 wards performed RDT diagnostic testing, 6823 HFs (85%) across 3063 wards offered antenatal services, whilst no HFs were found across 201 wards (Fig. 1). When the completeness and consistency of the reports were assessed, the laboratory reports from 1208 (15.3%) HFs across 141 (4.5%) wards and antenatal reports from 684 HFs (10.0%) across 70 (2.3%) wards were excluded from the analysis (Figs. 1 and 2a).

These HFs had either less than 50% reporting rate (RR), more than 5 consecutive months of missing reports or reports with extreme outliers. The overall proportion of extreme outliers was low with only 0.2% and 0.1% of total reports from laboratory and ANC registers removed, respectively. The majority of the HFs after exclusion (86% of HFs submitting laboratory reports and 90% of HFs submitting ANC reports) had more than 75% RR across the 36-month period of analysis with only 14% (across 20 wards) and 10% (across 104 wards) of the HFs with RR between 50 and 75% for laboratory and ANC reports.

Of the selected HFs used for stratification (n = 7098) (Fig. 2b), those offering both ANC and laboratory services accounted for 80.5% of all HFs, while 13.5% offering only laboratory services and 6% offering only ANC services. As a result, there were differing numbers of malaria indicators across the wards. Precisely, 2891 (87.3%) wards had all three routine indicators, 72 (2.2%) wards had only two indicators of RDT TPR and API, while 102 (3.1%) wards had only one indicator of ANC TPR. Excluded HFs with poor RR also accounted for some of the differing numbers of indicators across wards [143 (83%) wards with only one or two indicators and 41 (16%) wards with no facility points].

Data from the laboratory registers of the selected HFs were obtained for a total of 52.9 million malaria tests performed by *Pf*-pan RDT, of which 14.7 million were positive for malaria. Similarly, data from the ANC registers of the selected HFs were obtained for a total of 5.7 million malaria tests performed on pregnant women, of which 365,182 were tested positive for malaria (Fig. 1). When the distribution of the maximum annual mean values of all the indicators of wards within councils was examined, a heterogeneous distribution across wards was observed (Additional file 1: Fig. S6). For instance, in councils with *Pf*PR_5–16_ < 1%, the API ranged from 0 to 243 per 1000 populations, RDT TPR ranged from 0 to 76% and ANC TPR ranged from 0 to 10% across wards. The observed heterogeneity within the different councils confirmed the need for further characterizing malaria risk at the ward level.

### Risk categorization of councils using routine indicators

For the 2017 and 2019 surveys, estimates of malaria infection prevalence were available from a total of 693 sampled schools and 134,902 children across all 184 councils nationwide [63, 64]. During this period, the maximum of the annual mean council prevalence ranged from 0.0 to 85.0%. Following the allocation of councils to the four malaria risk strata, 38 councils (20.6%) had *Pf*PR_5–16_ < 1% (very low risk stratum), 32 councils (17.4%) had *Pf*PR_5–16_ 1 to < 5% (low risk stratum), 52 councils (28.3%) had *Pf*PR_5–16_ 5 to < 30% (moderate risk stratum) whilst 62 councils (33.7%) had *Pf*PR_5–16_ ≥ 30% (high risk stratum).

**Fig. 3:**
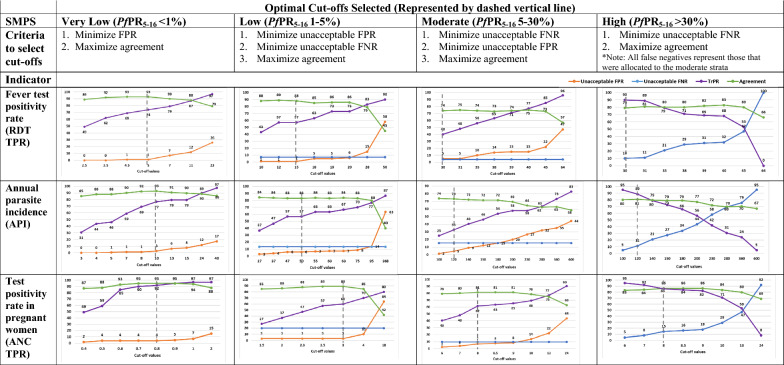
Misclassification analysis to select cut-offs for risk categories for the malaria indicators. *FPR* false positivity rate, *FNR* false negativity rate, *TrPR* true positivity rate, *TPR* test positivity rate

For each school prevalence risk group, the sensitivity, specificity and overall agreement for the different values of the routine indicator cut-offs are presented in Fig. 3. A total of two, four and six councils with *Pf*PR_5–16_ > 1% were misallocated into the very low strata for the selected cut-offs of RDT TPR, API and ANC TPR, respectively, which translated to an overall agreement of 93% for RDT TPR and API, and of 95% for ANC TPR. Similarly, for the selected low category cut-offs of RDT TPR, API and ANC TPR, a total of two, seven and four councils, respectively, with *Pf*PR_5–16_ > 5% were misallocated to the low strata whilst maintaining the overall proportion agreement between indicators at 88% for RDT TPR and ANC TPR and 83% for API. When selecting the optimal cut-off to define the moderate and high categories for the routine indicators, a total of two, seven and four councils with *Pf*PR_5–16_ 5 to < 30% were misallocated into the low or very low strata for the selected cut-offs of RDT TPR, API and ANC TPR, respectively. No councils belonging to the high risk group of *Pf*PR_5–16_ ≥ 30% were misallocated to low and very low risk group by the selected routine indicator cut-offs.

Table 2 summarizes the final selected cut-offs derived from the misclassification analysis conducted at the council level, and subsequently applied to categorize each of the routine indicators per ward into the four risk groups.

**Table 2:** Selected routine indicator cut-offs to categorize these indicators into risk groups at ward level

Prevalence in school children	Very low risk (*Pf*PR_5–16_ < 1%)	Low risk (*Pf*PR_5–16_ 1 to < 5%)	Moderate risk (*Pf*PR_5–16_ 5 to < 30%)	High risk (*Pf*PR_5–16_ ≥ 30%)
Laboratory-based results				
1. Fever test positivity rate	< 5	5 to < 15	15 to < 30	≥ 30
2. Annual parasite incidence	< 10	10 to < 50	50 to < 120	≥ 120
Antenatal clinic results				
3. Test positivity rate	< 0.8	0.8 to < 3	3 to < 8	≥ 8

The corresponding spatial distribution by ward for each of the malaria risk indicator using the selected cut-offs is summarized in Additional file 1: Fig. S7. Although variations exist between indicators in terms of the number of wards falling within each risk category, overall a similar pattern of heterogeneity was observed. The wards in the northwest and southeast of the country were mostly categorized into the moderate to high risk groups, while the wards in the central corridor running from northeast to southwest were mostly in the low and very low risk groups consistently across the three routine indicators.

### Micro-stratification of wards and malaria risk heterogeneity

**Fig. 4:**
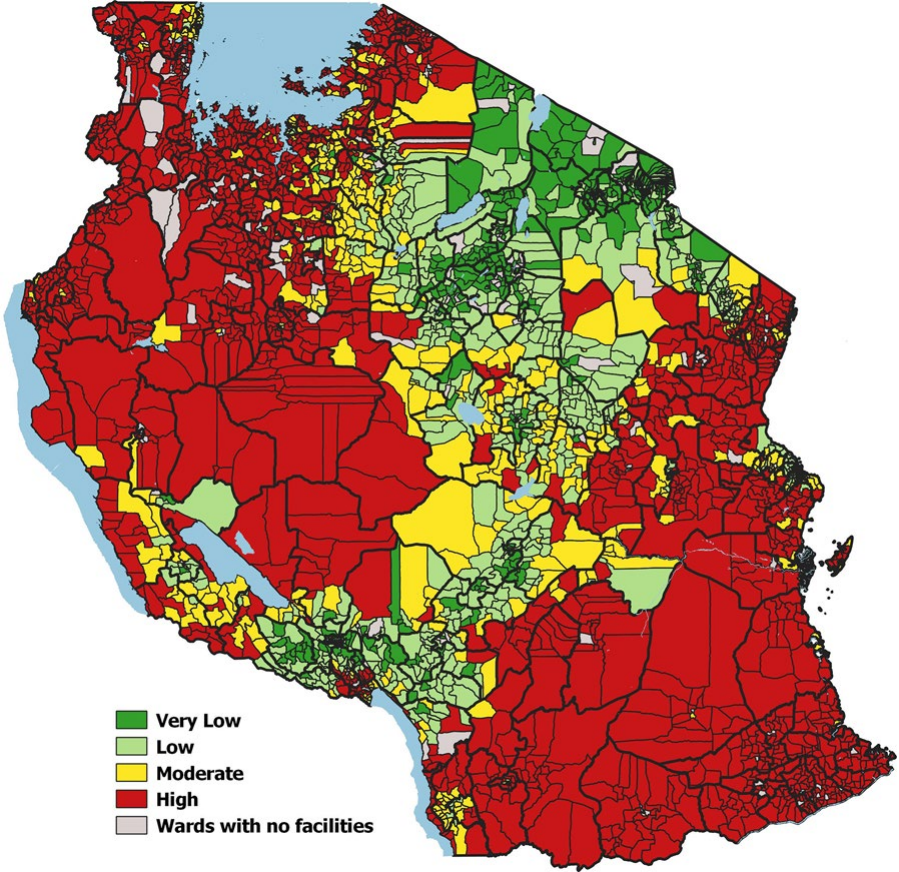
Micro-stratification of malaria risk in mainland Tanzania for the period 2017–2019

The resulting micro-stratification following the combination of multiple malaria routine indicators is shown in Fig. 4.

In total, 10.5% of the population resided in the 353 wards allocated to the very low strata, 28.6% resided in the 717 wards allocated to the low strata, 16.1% resided in the 525 wards allocated to the moderate strata, and 39.8% resided in the 1470 wards allocated to the high strata. The 246 wards with no HFs represented approximately 5% of the total country population and because of the lack of all routine malaria indicators, no stratification could be conducted there.

**Fig. 5:**
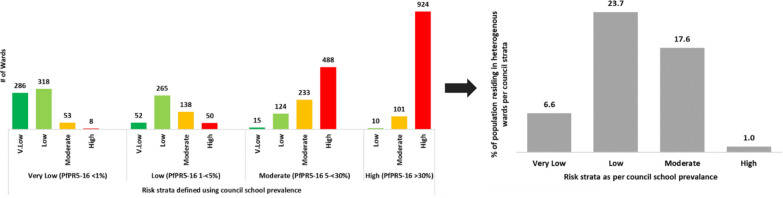
Number of heterogeneous wards per council prevalence risk group and corresponding population (%) residing in these wards

The micro-stratification process revealed varying levels of heterogeneity within the wards of 80 councils (Fig. 5; Additional file 2: Table S3).

Of the councils with very low (*Pf*PR_5–16_ < 1%) and low (*Pf*PR_5–16_ 1 to < 5%) prevalence, 12 had 6.6% of the population residing across 61 wards in the moderate-high transmission strata and 30 had 23.7% of the population residing across 188 wards in the moderate-high strata. Similarly, of the councils with moderate (*Pf*PR_5–16_ 5 to < 30%) and high (*Pf*PR_5–16_ > 30%) prevalence, 32 had 17.6% of the population residing across the 139 wards in very low-low transmission strata and 6 had 1% of the population residing in the 10 wards with low transmission strata. Overall, councils with low prevalence had the highest proportion of heterogeneous wards (37.2%), followed by councils with moderate prevalence (16.2%), then by councils with very low prevalence (9.2%) and finally the councils with high prevalence (1%).

## Discussion

This paper demonstrates at the level of an entire country the potential of using quality routine malaria indicators in informing on the malaria risk at the more granular levels: the third administrative level (wards). It builds on previous efforts taken by mainland Tanzania in using routine malaria indicators to stratify malaria risk at the second administrative level (councils) [37].

A strong feature of the method presented here is the triangulation of information from multiple malariometric indicators. The selected routine indicators represented a valuable and rich source of data in space and time across different age and immunological groups (children *versus* all ages and pregnant women). The approach categorized the three selected routine indicators using school prevalence classifications as a gold standard, since the prevalence rate in children is widely used as a reference metric for defining malaria risk [13, 68]. Because of the sampling strategy used in Tanzania for school surveys, it added further confidence to school prevalence serving as an appropriate benchmark for the misclassification analysis. Furthermore, the misclassification analysis was conservative and inclined to allocating wards to higher strata than to the lower strata that would otherwise receive reduced control efforts.

The use of routine indicators was contingent on the availability of data. Using data from HFs with RR > 50% ensured the reliability of our estimates. Applying a higher threshold for RR would have meant that only a small proportion of HFs (~ 20–25%) could be included in the analysis. Hence, the criteria of 50% reporting represented a good compromise between data quality and the number of HF data available for analysis (Additional file 1: Fig. S8). Current guidelines by WHO recommends assessing four core dimensions for understanding the quality of routine data. These include: (i) completeness and timeliness of data; (ii) internal consistency of reported data (presence of outliers, consistency over time and consistency between data elements); (iii) external consistency with other data sources; and, (iv) external comparison with population data [69]. Due to the limited elements reported within the laboratory registers of Tanzania, the consistency with other data elements was not possible. Generally, the RR for HFs data were high in Tanzania with only a small proportion of reports having extreme outliers, allowing the use of such data in this systematic way for risk assessment. The country has also recently launched the malaria service and data quality improvement tool that involves conducting facility supervision by council health teams on a quarterly basis to assess the malaria related services and data quality performance [70].

Although this may not be the case in other countries in SSA and could limit the applicability of this approach elsewhere, it stresses the importance for other countries to work towards strengthening their routine information system and reporting practices. Furthermore, the work presented in this paper made use of the local data available in Tanzania, as such, the approach would need to be tailored in other countries according to available metrics and local context.

The resulting risk map detailed to ward level (Fig. 4) revealed significant heterogeneity in malaria risk within 80 councils and helped to identify areas where the population could be further prioritized to receive more targeted community-based interventions. For instance, Bumbuli District Council is currently in the very low transmission strata, but the micro-stratification process revealed wards in the moderate and high transmission that could qualify for increased long-lasting insecticidal nets (LLIN) distribution (Additional file 1: Fig. S9). Compared to previous approaches of distributing LLINs universally across all wards [71], this new knowledge could finely target LLIN distribution within such wards, allowing a more efficient allocation of resources within a council that was previously assumed to have a uniform risk.

Supporting ministries of health to establish a quantitatively and qualitatively high-performance routine surveillance system, and strengthening the ability of NMCPs to analyse these data for developing stratification risk maps and on from that for decision making, is imperative for more efficient malaria control [1, 72]. It is crucial that each malaria-endemic country’s capacity is strengthened with regard to reliable data collection, detection of data biases, and its ability for conducting sensible analysis on a routine basis. Increased usage of maps for local decision making by NMCPs promotes knowledge and understanding of the various data sources and their limitations, trust and perceived ownership of the data, and finally increased knowledge and understanding of the processes of map construction [14].

The work presented here has some limitations that future work might address. The use of crude estimates of routine data does not account for important factors such as treatment-seeking rates, temporal and spatial missingness in data, the underlying heterogeneous distribution of the population and the differing testing rates between transmission settings, all of which can potentially under/over-estimate positivity rates [51, 73]. There have been many recent advances in statistical tools that use spatiotemporal modelling and imputation methods to better handle incomplete data and account for important biases present in routine data [32, 35, 43]. Since these approaches are complex, future work may explore comparing crude routine estimates against more complex statistical data modelling, in order to find an optimum point between accuracy and local ability to handle the data analysis process.

The estimates of the routine indicators used in the present analysis come with uncertainty due to sampling error (Additional file 1: Fig. S10). The risk strata assigned to each ward through the approach described in this paper did not account for this uncertainty. Thus the uncertainty in the micro-stratification risk strata was quantified at the ward level. First, the uncertainty of the individual routine indicator estimates, measured using the standard error, were obtained using multilevel regression analysis and then a sampling-based approach was used to estimate the probability of being in each risk strata for each ward (Additional file 1: Text S3). The results of the regression and sampling-based analysis (Additional file 1: Text S4, Fig. S12) highlight the importance of considering the variation of indicators when conducting the micro-stratification, and in estimating the certainty of the assigned risk strata. While for the majority of wards (over 60%), considering the variability of indicators did not change the assigned risk stratum, a substantial proportion of wards were more sensitive to the uncertainty in the estimated indicators. These wards had a reasonable probability of being assigned to the risk stratum immediately below that of the initially assigned stratum.

Although the micro-stratification approach adopted by the NMCP in Tanzania was more conservative, ensuring that wards were not misallocated to the lower strata, which would receive fewer vector control interventions, it is important that NMCPs take this uncertainty into account for more efficient planning of interventions. Specifically, the wards with a low assignment probability would require more careful investigation of the possible causes of the greater uncertainty in the estimated indicators. If the uncertainty is partly due to increased transmission heterogeneity, this would suggest that a localized deployment of interventions would be more appropriate compared to a ward-level approach. However, if the uncertainty is due to data collection and reporting, then more efforts need to be channeled towards optimizing the collection procedures.

Obviously, HFs may not always reflect the actual transmission status of the ward since people from surrounding wards may also utilize their services. Furthermore, the estimates may not always represent the universe of all HFs since poor performing HFs and private providers that are not linked to the DHIS2 are not captured without further adaptations. Obtaining accurate estimates of population denominators is currently a major challenge for defining HF catchment areas [74] in view of computing incidence rates, and until this knowledge is made available, the use of existing operational administrative boundaries as a proxy will continue to serve as the reference guide.

The current micro-stratification only considered the maximum value of the annual estimates for all ages in the past 3 years from DHIS2. It may be important to overlay the epidemiological risk map with other layers of information that are known to affect transmission such as urbanization, seasonality, monthly trends, disaggregation by age groups, marginalization, intervention coverage, ecological factors as well as socio-economic and population factors.

Furthermore, the availability of a comprehensive list of geo-coded HFs through the master HF list, that is dynamically updated in the HMIS/DHIS2, is a challenge in many parts of SSA [75, 76]. Ideally, the DHIS2 should represent information from all healthcare providers, however this is often not the case in many countries, with a large proportion of HFs missing in the DHIS2. Availability of an updated list of health providers is crucial to allow understanding of true reporting completeness, and availability of its geo-coded information allows linkage of HFs to its correct administrative boundaries especially at the finer spatial scales for correct quantification of risks. Efforts are needed to encourage countries to geo-reference all HFs and accordingly update their national databases.

Finally, the work presented here did not account for the fact that the relationship between the different indicators that represent different population age groups may not always be linear. An in-depth understanding of how they relate to one another and with more traditional measures of modelled prevalence estimates in the different transmission settings is crucial. Efforts to understand this relationship and incorporate routine data sources into modelled prevalence risk maps are emerging [77].

The WHO High Burden to High Impact (HBHI) strategy recommends countries to conduct stratification analysis at the sub-national levels, preferably at district level or at lower levels in accordance with the local context [78]. Mainland Tanzania has fully adopted a sub-national tailoring of interventions at the council level [57]. It is now recognizing the need for micro-stratification and decentralization of malaria control as indicated in its current strategic plan [57]. Wards are expected to become the ultimate target for further evidence-based malaria control planning by the Council Health Management Teams (CHMTs), especially for community-based interventions including community case management and focal vector control initiatives such as indoor residual spraying (IRS) and larviciding, down to ward level. Macro-stratification becomes more relevant across councils with homogenous transmission that require universal allocation of interventions across its population. However, for those councils with heterogeneous transmission within its administrative boundaries, these would need concentrated efforts in areas that most need them. The role of CHMTs in highly malaria-endemic countries has been traditionally limited to the operationalization at council level of key preventative malaria interventions such as LLINs, IRS, case management, and intermittent preventative treatment in pregnant women (IPTp), planned at central levels.

Whether the operationalization of micro-stratification and micro-planning is feasible remains to be assessed and will require close monitoring of the processes at all levels to ensure that it is replicated across councils. More importantly, there is a growing need to capacitate CHMTs to assemble, clean, interpret, and understand associated levels of uncertainty in their local data so as to undertake assessments of the local heterogeneity especially of wards that are not transitioning its transmission levels downwards at the same rate as others. For this, the need for granular data is crucial to empower the CHMTs to make use of the local data across health sectors. Micro-stratification is expected to allow this profound change in health planning processes by promoting a culture of data usage and equip council level with the capacity and tools to understand and appropriately respond to the local situation.

## Conclusion

The micro-stratification approach undertaken for mainland Tanzania has moved the agenda from council-level risk mapping to one at ward level reflecting the need for the decentralization of malaria control planning. Continuous efforts to improve routine data remains crucial for ensuring a reliable source of data for local epidemiological monitoring at sub-council level. This can have immediate potential in capacitating CHMTs to take charge of their routine data and respond in an appropriate manner to maximize impact and turn malaria surveillance into a core intervention.

## Supplementary Information

**Additional file 1.** Additional tables, text and figures.

**Additional file 2: Table S3.** Shows the proportion of heterogeneity per council.

## Data Availability

Data from routine HMIS/DHIS2 are not publicly available and were obtained with request from the National Malaria Control Programme of mainland Tanzania. Restrictions apply to the availability of these data and permission can be obtained with reasonable request from the Ministry of Health of mainland Tanzania.

## Abbreviations

ANC: Antenatal care
API: Annual parasite incidence
CHMT: Council Health Management Team
DHIS2: District Health Information Software
FPR: False positivity rate
FNR: False negativity rate
GTS: Global technical strategy
HBHI: High Burden to High Impact
HF: Health facilities
HMIS: Health Management Information System
IPTp: Intermittent preventative treatment in pregnant women
IRS: Indoor residual spraying
LLIN: Long-lasting insecticidal nets
MIS: Malaria Indicator Survey
MoH: Ministry of Health
RDT: Malaria rapid diagnostic testing
NBS: National Bureau of Statistics
NMCP: National Malaria Control Programme
NMSP: National Malaria Strategic Plan
*Pf*PR: *Plasmodium falciparum* prevalence rate
RR: Reporting rates
SMMSP: Supplementary Mid-term Malaria Strategic Plan
SMPS: School malaria parasitaemia survey
SSA: Sub-Saharan Africa
TPR: Test positivity rate
TrPR: True positivity rate
UDSM: University of Dar es Salaam
WHO: World Health Organization

## References

[1] WHO, RBM. High burden to high impact: a targeted malaria response. Geneva: World Health Organization, and RBM Partnership to End Malaria; 2018. https://www.who.int/malaria/publications/atoz/high-impact-response/en/.

[2] WHO. Global technical strategy for malaria 2016–2030. Geneva: World Health Organization; 2015. https://www.who.int/malaria/publications/atoz/9789241564991/en/.

[3] Pampana EJ, Russell PF (1955). Malaria: a world problem. Chron World Health Organ.

[4] Boyd MF (1949). Malariology. A comprehensive review of all aspects of this group of diseases from a global standpoint.

[5] Lysenko AY, Semashko IN (1968). Geography of malaria: a medico-geographic profile of an ancient disease. Itogi Nauk Med Geogr.

[6] Snow RW, Sartorius B, Kyalo D, Maina J, Amratia P, Mundia CW (2017). The prevalence of *Plasmodium falciparum* in sub-Saharan Africa since 1900. Nature.

[7] Snow RW, Noor AM. Malaria risk mapping in Africa: the historical context to the Information for Malaria (INFORM) project. Working Paper in support of the INFORM Project funded by the Department for International Development and the Wellcome Trust, Nairobi, Kenya; 2015.

[8] Diggle PJ, Tawn JA, Moyeed RA (1998). Model-based geostatistics. Appl Stat.

[9] Giorgi E, Diggle P, Snow RW, Noor AM (2018). Geostatistical methods for disease mapping and visualisation using data from spatio-temporally referenced prevalence surveys. Int Stat Rev.

[10] Odhiambo JN, Kalinda C, Macharia PM, Snow RW, Sartorius B (2020). Spatial and spatio-temporal methods for mapping malaria risk: a systematic review. BMJ Glob Health.

[11] Noor AM, Kinyoki DK, Mundia CW, Kabaria CW, Wambua JM, Alegana VA (2014). The changing risk of *Plasmodium falciparum* malaria infection in Africa: 2000–10: a spatial and temporal analysis of transmission intensity. Lancet.

[12] Bhatt S, Weiss DJ, Cameron E, Bisanzio D, Mappin B, Dalrymple U (2015). The effect of malaria control on *Plasmodium falciparum* in in Africa between 2000 and 2015. Nature.

[13] Weiss DJ, Lucas TCD, Nguyen M, Nandi AK, Bisanzio D, Battle KE (2019). Mapping the global prevalence, incidence, and mortality of *Plasmodium falciparum*, 2000–17: a spatial and temporal modelling study. Lancet.

[14] Ghilardi L, Okello G, Nyondo-Mipando L (2020). How useful are malaria risk maps at the country level? Perceptions of decision-makers in Kenya, Malawi and the Democratic Republic of Congo. Malar J.

[15] Noor AM, ElMardi KA, Abdelgader TM, Patil AP, Amine AAA, Bakhiet S (2012). Malaria risk mapping for control in the Republic of Sudan. Am J Trop Med Hyg.

[16] Noor AM, Gething PW, Alegana VA, Patil AP, Hay SI, Muchiri E (2009). The risks of malaria infection in Kenya in 2009. BMC Infect Dis.

[17] Chipeta MG, Giorgi E, Mategula D, Macharia PM, Ligomba C, Munyenyembe A (2019). Geostatistical analysis of Malawi’s changing malaria transmission from 2010 to 2017. Wellcome Open Res.

[18] Raso G, Schur N, Utzinger J, Koudou BG, Tchicaya ES, Rohner F (2012). Mapping malaria risk among children in Côte d’Ivoire using Bayesian geo-statistical models. Malar J.

[19] Giorgi E, Osman AA, Hassan AH, Ali AA, Ibrahim F, Amran JGH (2018). Using non-exceedance probabilities of policy-relevant malaria prevalence thresholds to identify areas of low transmission in Somalia. Malar J.

[20] Kang SY, Battle KE, Gibson HS, Ratsimbasoa A, Randrianarivelojosia M, Ramboarina S (2018). Spatio-temporal mapping of Madagascar’s malaria indicator survey results to assess *Plasmodium falciparum* endemicity trends between 2011 and 2016. BMC Med.

[21] Macharia PM, Giorgi E, Noor AM, Waqo E, Kiptui R, Okiro EA (2018). Spatio-temporal analysis of *Plasmodium falciparum* prevalence to understand the past and chart the future of malaria control in Kenya. Malar J.

[22] Semakula M, Niragire FI, Faes C (2020). Bayesian spatio-temporal modeling of malaria risk in Rwanda. PLoS ONE.

[23] Ssempiira J, Nambuusi B, Kissa J, Agaba B, Makumbi F, Kasasa S (2017). The contribution of malaria control interventions on spatio-temporal changes of parasitaemia risk in Uganda during 2009–2014. Parasites Vectors.

[24] Yankson R, Anto EA, Chipeta MG (2019). Geostatistical analysis and mapping of malaria risk in children under 5 using point-referenced prevalence data in Ghana. Malar J.

[25] Githinji S, Oyando R, Malinga J, Ejersa W, Soti D, Rono J (2017). Completeness of malaria indicator data reporting via the District Health Information Software 2. Malar J.

[26] Maina JK, Macharia PM, Ouma PO, Snow RW, Okiro EA (2017). Coverage of routine reporting on malaria parasitological testing in Kenya, 2015–2016. Glob Health Action.

[27] Chilundo B, Sundby J, Aanestad M (2004). Analysing the quality of routine malaria data in Mozambique. Malar J.

[28] WHO. Scaling up diagnostic testing, treatment and surveillance for malaria. Geneva: World Health Organization; 2012. https://www.who.int/malaria/publications/atoz/test_treat_track_brochure.pd.

[29] Dehnavieh R, Haghdoost A, Khosravi A, Hoseinabadi F, Rahimi H, Poursheikhali A (2018). The district health information system (DHIS2): a literature review and metasynthesis of its strengths and operational challenges based on the experiences of 11 countries. Health Inf Manag.

[30] WHO. Data quality review: a toolkit for facility data quality assessment. Module 1. Framework and metrics. Geneva: World Health Organization; 2020. https://www.who.int/data/data-collection-tools/health-service-data/data-quality-assurance-dqa.

[31] Ashton RA, Bennett A, Yukich J, Bhattarai A, Keating J, Eisele TP (2017). Methodological considerations for use of routine health information system data to evaluate malaria program impact in an era of declining malaria transmission. Am J Trop Med Hyg.

[32] Alegana VA, Okiro EA, Snow RW (2020). Routine data for malaria morbidity estimation in Africa: challenges and prospects. BMC Med.

[33] Arambepola R, Keddie SH, Collins EL, Twohig KA, Amratia P, Bertozzi-Villa A (2020). Spatiotemporal mapping of malaria prevalence in Madagascar using routine surveillance and health survey data. Sci Rep.

[34] Awine T, Malm K, Peprah NY, Silal SP (2018). Spatio-temporal heterogeneity of malaria morbidity in Ghana: analysis of routine health facility data. PLoS ONE.

[35] Bennett A, Yukich J, Miller JM, Vounatsou P, Hamainza B, Ingwe MM (2014). A methodological framework for the improved use of routine health system data to evaluate national malaria control programs: evidence from Zambia. Popul Health Metr.

[36] Kigozi SP, Kigozi RN, Sebuguzi CM, Cano J, Rutazaana D, Opingo J (2020). Spatial-temporal patterns of malaria incidence in Uganda using HMIS data from 2015 to 2019. BMC Public Health.

[37] Thawer SG, Chacky F, Runge M, Reaves E, Mandike R, Lazaro S (2020). Sub-national stratification of malaria risk in mainland Tanzania: a simplified assembly of survey and routine data. Malar J.

[38] Alegana VA, Suiyanka L, Macharia PM, Ikahu-Muchangi G, Snow RW (2021). Malaria micro-stratification using routine surveillance data in Western Kenya. Malar J.

[39] Afrane YA, Zhou G, Githeko AK, Yan G (2013). Utility of health facility-based malaria data for malaria surveillance. PLoS ONE.

[40] Oduro AR, Bojang KA, Conway DJ, Corrah T, Greenwood BM, Schellenberg D (2011). Health centre surveys as a potential tool for monitoring malaria epidemiology by area and over time. PLoS ONE.

[41] Carter R, Mendis KN, Roberts D (2000). Spatial targeting of interventions against malaria. Bull World Health Organ.

[42] Woolhouse MEJ, Dye C, Etard JF, Smith T, Charlwood JD, Garnett GP (1997). Heterogeneities in the transmission of infectious agents: implications for the design of control programs. Proc Natl Acad Sci USA.

[43] Sturrock HJW, Bennett AF, Midekisa A, Gosling RD, Gething PW, Greenhouse B (2016). Mapping malaria risk in low transmission settings: challenges and opportunities. Trends Parasitol.

[44] Mogeni P, Omedo I, Nyundo C, Kamau A, Noor A, Bejon P (2017). Effect of transmission intensity on hotspots and micro-epidemiology of malaria in sub-Saharan Africa. BMC Med.

[45] Bousema T, Griffin JT, Sauerwein RW, Smith DL, Churcher TS, Takken W (2012). Hitting hotspots: spatial targeting of malaria for control and elimination. PLoS Med.

[46] WHO (2018). Malaria surveillance, monitoring & evaluation: a reference manual.

[47] Omumbo JA, Noor AM, Fall IS, Snow RW (2013). How well are malaria maps used to design and finance malaria control in Africa?. PLoS ONE.

[48] Lindblade KA, Li XH, Galappaththy GL, Noor A, Kolaczinski J, Alonso PL (2019). Country-owned, country-driven: perspectives from the World Health Organization on malaria elimination. Methods Mol Biol.

[49] Byrne E, Saebø JI (2022). Routine use of DHIS2 data: a scoping review. BMC Health Ser Res.

[50] Etamesor S, Ottih C, Salihu IN (2018). Data for decision making: using a dashboard to strengthen routine immunisation in Nigeria. BMJ Glob Health.

[51] Maïga A, Jiwani SS, Mutua MK, Porth TA, Taylor CM, Asiki G (2019). Generating statistics from health facility data: the state of routine health information systems in Eastern and Southern Africa. BMJ Glob Health.

[52] National Malaria Control Programme (NMCP). Mid-term review report of national malaria strategic plan 2015–2020. Dar es Salaam, Tanzania; 2017.

[53] National Malaria Control Programme (NMCP). Consultative malaria expert meeting report 2018. Ministry of Health, Community Development, Gender, Elderly and Children. Dar es Salaam, Tanzania; 2018.

[54] Runge M, Molteni F, Mandike R, Snow RW, Lengeler C, Mohamed A (2020). Applied mathematical modelling to inform national malaria policies, strategies and operations in Tanzania. Malar J.

[55] Runge M, Snow RW, Molteni F, Thawer S, Mohamed A, Mandike R (2020). Simulating the council-specific impact of anti-malaria interventions: a tool to support malaria strategic planning in Tanzania. PLoS ONE.

[56] National Malaria Control Programme (NMCP). Supplementary midterm malaria strategic plan 2018–2020. Ministry of Health, Community Development, Gender, Elderly and Children. Tanzania, Dar es Salaam; 2018.

[57] National Malaria Control Programme (NMCP). Malaria strategic plan 2021–2025. Ministry of Health, Community Development, Gender, Elderly and Children. Tanzania, Dar es Salaam; 2021.

[58] National Bureau of Statistics (Tanzania), Office of Chief Government Statistician (Zanzibar). 2012 population and housing census. Dar es Salaam; 2013.

[59] Kahama-Maro J, D'Acremont V, Mtasiwa D, Genton B, Lengeler C (2011). Low quality of routine microscopy for malaria at different levels of the health system in Dar es Salaam. Malar J.

[60] Dancho M, Vaughan D. anomalize: tidy anomaly detection. 2020. https://CRAN.R-project.org/package=anomalize.

[61] http://hfrportal.moh.go.tz/.

[62] Chacky F, Runge M, Rumisha SF, Machafuko P, Chaki P, Massaga JJ (2018). Nationwide school malaria parasitaemia survey in public primary schools, the United Republic of Tanzania. Malar J.

[63] Ministry of Health, Community Development, Gender, Elderly and Children and the National Malaria Control Programme. School malaria and nutrition survey (SMNS) report. Tanzania, Dar es Salaam; 2021.

[64] Ministry of Health, Community Development, Gender, Elderly and Children and the National Malaria Control Programme. School malaria parasitaemia survey (SMPS) report. Tanzania, Dar es Salaam; 2019.

[65] WHO. Global malaria programme. A framework for malaria elimination. Geneva: World Health Organization; 2017. http://apps.who.int/iris/bitstream/10665/254761/1/9789241511988-eng.pdf.

[66] Team Rs. RStudio: integrated development for R. RStudio, PBC. Boston, MA URL; 2020. http://www.rstudio.com/.

[67] Team QGISD. QGIS geographic information system. Open source geospatial foundation project. http://qgis.osgeo.org.

[68] Alegana VA, Macharia PM, Muchiri S, Mumo E, Oyugi E, Kamau A (2021). *Plasmodium falciparum* parasite prevalence in East Africa: updating data for malaria stratification. PLOS Glob Public Health.

[69] WHO (2017). Data quality review: module 2 desk review of data quality.

[70] National Malaria Control Programme (NMCP). Operational manual for implementing malaria services and data quality improvement (MSDQI). Ministry of Health, Community Development, Gender, Elderly and Children. Dar es Salaam, Tanzania; 2017.

[71] Renggli S, Mandike R, Kramer K, Patrick F, Brown NJ, McElroy PD (2013). Design, implementation and evaluation of a national campaign to deliver 18 million free long-lasting insecticidal nets to uncovered sleeping spaces in Tanzania. Malar J.

[72] Boerma T, Mathers C (2015). The World Health Organization and global health estimates: improving collaboration and capacity. BMC Med.

[73] Amboko B, Stepniewska K, Macharia P, Machini B, Bejon P, Snow RW (2020). Trends in health workers’ compliance with outpatient malaria case-management guidelines across malaria epidemiological zones in Kenya, 2010–2016. Malar J.

[74] Macharia PM, Ray N, Giorgi E (2021). Defining service catchment areas in low-resource settings. BMJ Glob Health.

[75] USAID, World Health Organization (2018). Master facility list resource package: guidance for countries wanting to strengthen their MFL.

[76] Maina J, Ouma PO, Macharia PM, Alegana VA, Mitto B, Fall IS (2019). A spatial database of health facilities managed by the public health sector in sub Saharan Africa. Sci Data.

[77] Yukich J, Briët O, Bretscher MT, Bennett A, Lemma S, Berhane Y (2012). Estimating *Plasmodium falciparum* transmission rates in low-endemic settings using a combination of community prevalence and health facility data. PLoS ONE.

[78] WHO (2020). Technical brief for countries preparing malaria funding requests for the Global Fund (2020–2022).

