# Supplementary material for: The use of routine health facility data for micro-stratification of malaria risk in mainland Tanzania
Source: Malar J. 2022 Nov 18;21:345. doi: 10.1186/s12936-022-04364-7 (PMC9675286; doi:10.1186/s12936-022-04364-7)
Supplement: Supplementary file 1 — Additional file 1. Additional tables, text and figures. [file 12936_2022_4364_MOESM1_ESM.docx]

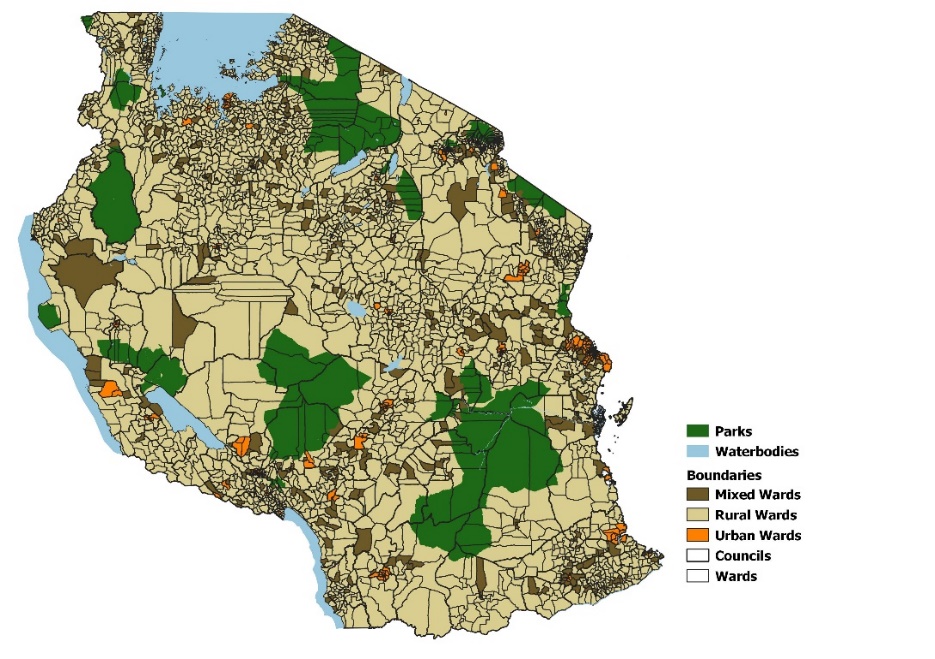


**Figure S1**: Administrative boundaries and distribution of urban, rural and mixed wards in mainland Tanzania.


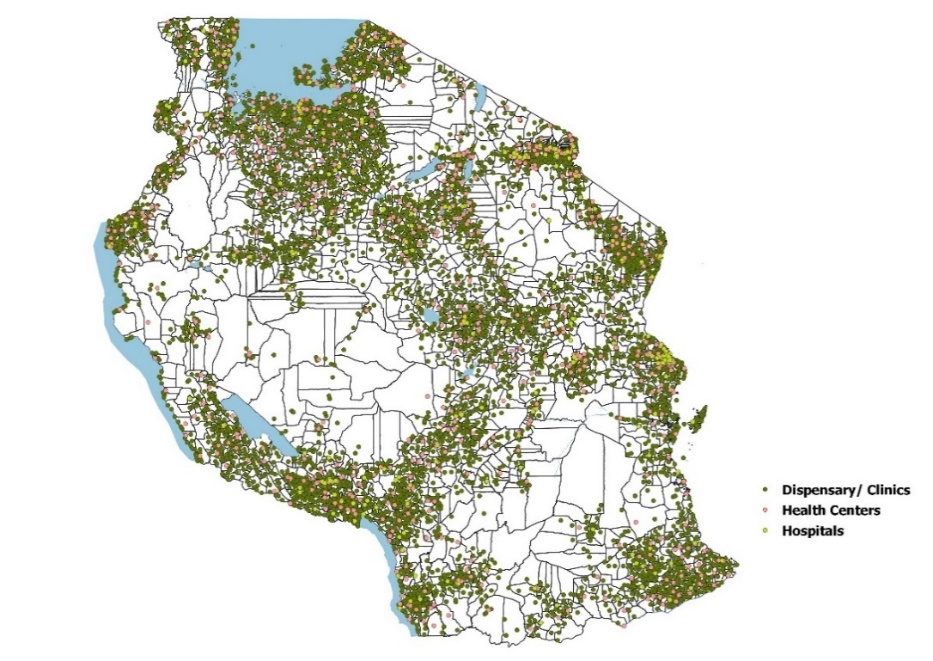


**Figure S2**: Location of health facilities (HFs) by type (n=7,988)

*For 157 (2%) of the total HFs, the ward name in the master HF list did not appear in the existing ward shape file and therefore the geo-coordinate was used to guide the ward location in the shape file. The geo coordinates for another 180 (2%) HFs could not be obtained from the master HF list and thus open source platforms such as Google Earth was used to retrieve the information.

**Text S1 Identification of outliers with the *anomalize* R package:**

Extreme outliers in the routine indicator values were defined as the monthly values that significantly deviated from the health facility’s (HF) overall time series trend across the 36 months. The R package *anomalize* performs the outlier detection on the remainder from a time series analysis after removing the seasonal and trend component [1]. Following decomposition of data through the “Twitter” approach, the inner quartile range of the data series was used to establish the distribution on the remainder. A factor of X9 was used to set the limits above and below the inner quartile range and any remainder beyond the limit was considered an extreme outlier. A visual inspection was done to verify the identified outliers and these were subsequently removed and treated as a missing monthly report.

**Reference**

1. Dancho M, Vaughan D. anomalize: Tidy Anomaly Detection. 2020. Available from: https://CRAN.R-project.org/package=anomalize

**Table S1:** Contingency table to compute sensitivity and specificity for each indicator at council level using prevalence categories in school children (*Pf*PR_5-16_) as the ground truth

|  | **Malaria Indicator** | | |
| --- | --- | --- | --- |
| ***Pf*PR_5-16_** |  | **Predicted Stratum** | **Other Strata** |
|  | **True Stratum** | True Positive | False Negative |
|  | **Other Strata** | False Positive | True Negative |

**Text S2: Misclassification Analysis**

**Definitions:**

*Overall Agreement:* proportion of councils that belong to a particular stratum as defined by both the school prevalence and the routine indicator i.e., the proportion of agreement between the indicators.

*Sensitivity or True Positivity Rate (TrPR)*: proportion of councils that belong to a particular stratum as defined by the school prevalence and were correctly classified into that stratum based on the routine malaria indicator.

*Specificity or True Negativity Rate (TrNR)*: proportion of councils that do not belong to a particular stratum as defined by the school prevalence and were correctly not allocated to that stratum based on the routine malaria indicator.

*False Positivity Rate (FPR)*: proportion of councils that do not belong to a particular stratum as defined by the school prevalence but were misclassified to that stratum based on the routine malaria indicator.

*False Negativity Rate (FNR)*: proportion of councils that belong to a particular stratum as defined by the school prevalence but were misclassified and not allocated to that stratum based on routine malaria indicator.

*Unacceptable False Positivity Rate (FPR)*: proportion of councils with *Pf*PR_5-16_ 1-<5%, *Pf*PR_5-16_ 5-30% and *Pf*PR_5-16_ >30% that do not belong to a particular stratum as defined by the school prevalence but were misclassified to a lower stratum based on the routine malaria indicator (Fig S3i).

*Unacceptable False Negativity Rate (FNR)*: proportion of councils with *Pf*PR_5-16_ 1-<5%, *Pf*PR_5-16_ 5-30% and *Pf*PR_5-16_ >30% that belong to a particular stratum as defined by the school prevalence but were misclassified to a lower stratum based on the routine malaria indicator (Fig S3ii).

| a.  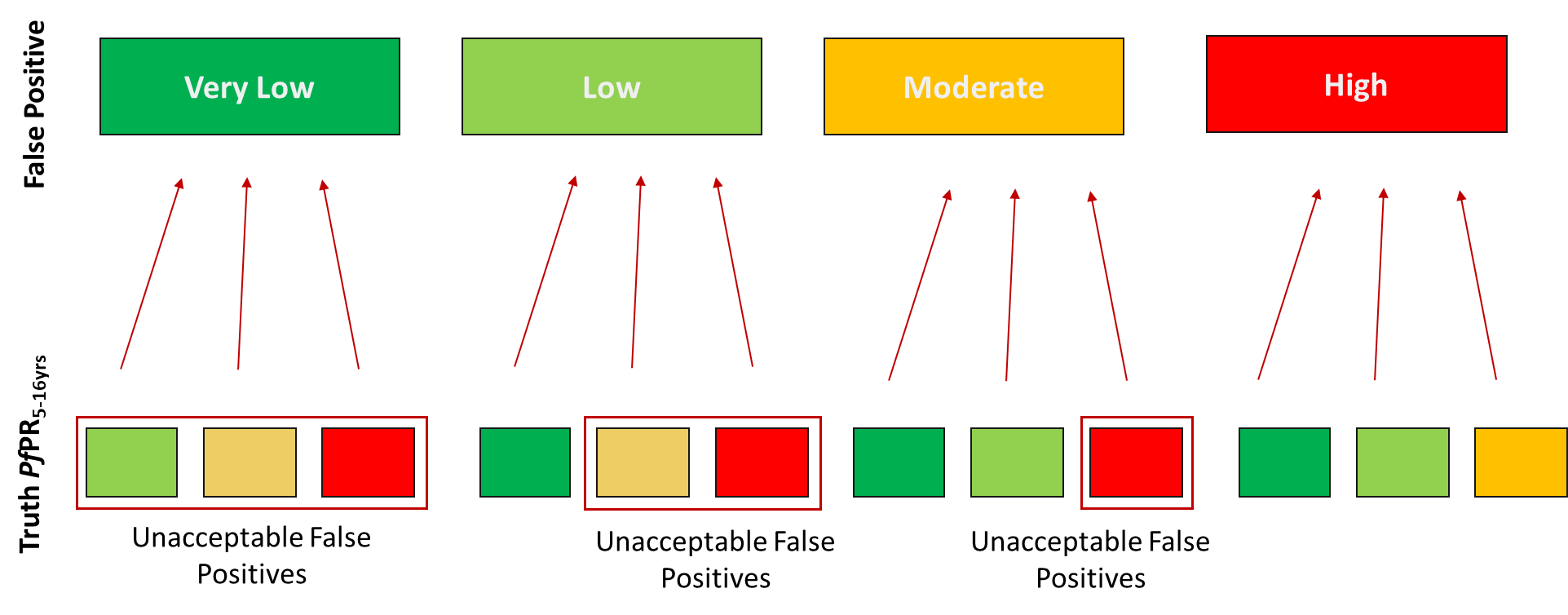 |
| --- |
| b.  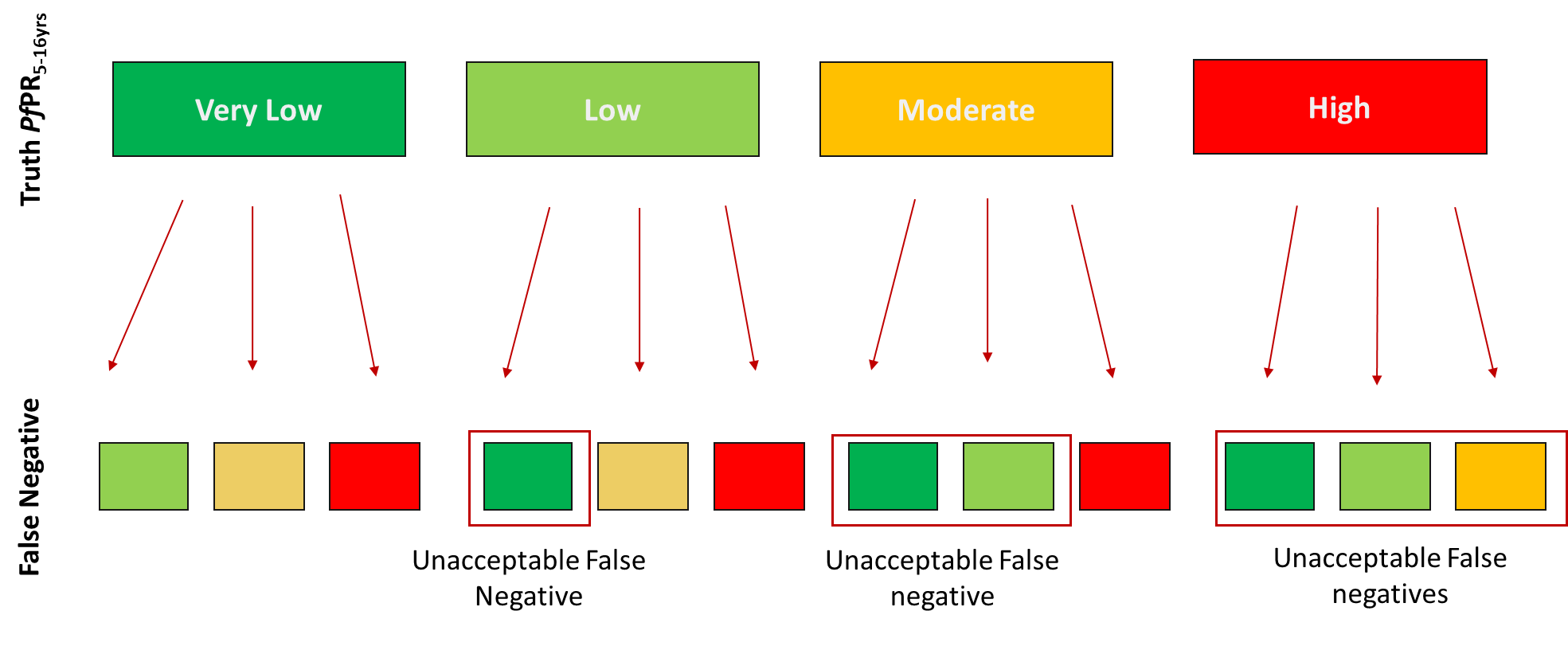 |

**Fig S3:** (i) Definition of unacceptable false positives per risk stratum, (ii) definition of unacceptable false negatives per risk stratum.

**Table S2:** Total score cut-offs depending on the number of indicators per ward

| **# of indicators** | **1** | **2** | **3** |
| --- | --- | --- | --- |
| **Very Low** | =1 | ≤2 | ≤3 |
| **Low** | =2 | 3-≤4 | 4-≤6 |
| **Moderate** | =3 | 5-≤6 | 7-≤9 |
| **High** | =4 | 7-≤8 | 10-≤12 |

**Text S3: Method for incorporating the uncertainty in ward-level routine indicator estimates in order to quantify the uncertainty in the risk stratification.**

**Estimating the standard errors of the routine indicator estimates:**

The risk strata per ward determined from the pragmatic approach did not account for the uncertainty in the routine indicator estimates. Thus, to quantify the uncertainty of the stratification of malaria risk at the ward level, the uncertainty of the individual routine indicator estimates was obtained using multilevel regression models. For each indicator and ward, a generalized linear mixed-effects regression model was defined with a random effect for HF. Precisely, a binomial logistic regression was defined for ANC TPR and mRDT TPR, whilst a Poisson regression was defined for the API. The standard errors of the regression coefficient estimates from the models were used to estimate the variation of the log- or logit- transformed indicators per ward. Subsequently, a sampling-based approach was used to evaluate the uncertainty of the risk strata for the wards.

In absence of information about the total number of HFs per ward and the ratio of the sampled HFs for routine indicators, a conservative assumption was adopted where we consider that the HFs were sampled from an infinite set. In Tanzania, the facility catchment population remains largely undefined. Therefore, to estimate the case incidence per facility, the ratio of facility Outpatient Department (OPD) attendance out of the total ward OPD attendance was used as a proxy to obtain the proportion of population residing within a facility’s catchment out of the total ward population. The regression model for the incidence of a HF i of a ward j (Y_ij_) was defined as follows:

$$Y_{ij} \sim Poisson(\lambda_{ij})$$

$$log\lambda_{ij}=\log({Pop}_{ij})+\beta_{j}+ \epsilon_{ij}$$

$\beta_{j}=\gamma_{00}+ u_{j}$

where:

λ_ij_ = incidence rate for HF i in ward j

Pop_ij_ = HF catchment population at risk for facility i in ward j

β_j_ = intercept of the dependent variable in ward j

ϵ_ij_ = residual error for HF i in ward j

u_j_ = random error component for the deviation of the intercept of a group from the overall intercept

Similarly, for ANC TPR and mRDT TPR of a HF i of a ward j (Y_ij_), binomial regression models with logit link functions were defined as:

$$Y_{ij} \sim Binomial(P_{ij})$$

$$logit\left( P_{ij} \right)=\beta_{j}+ \epsilon_{ij}$$

Where:

P_ij_ = positivity rate for HF i in ward j

β_j_ = intercept of the dependent variable in ward j

ϵ_ij_ = residual error

The performance of the three regression models was evaluated by inspecting the correlations of the observed values versus the model estimates (Fig S4).


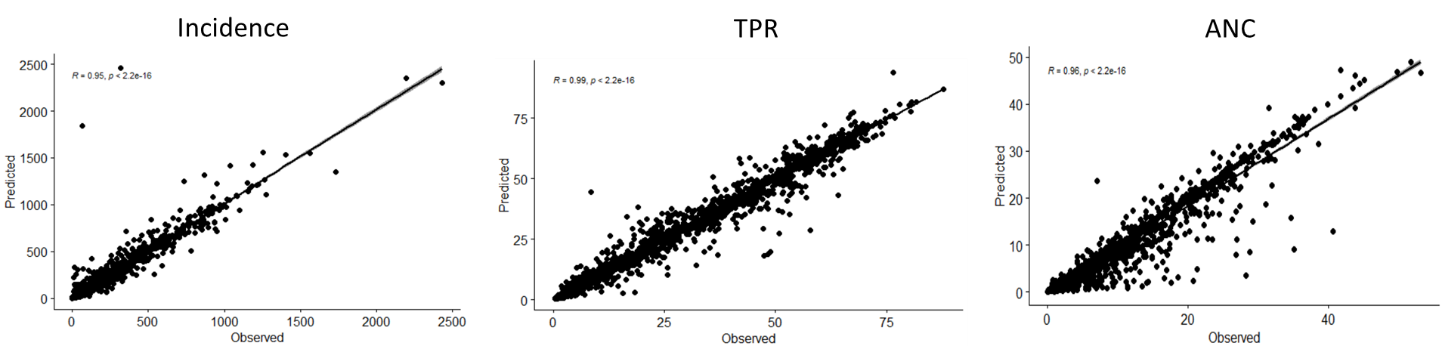


**Fig S4:** Scatter plots of the observed maximum ward indicator values vs the estimates obtained from the regression models.

The standard errors for the regression coefficient estimates from the models were used to define the variability of indicators per ward. The regression analysis could only be performed on wards with collected routine data from more than 1 HF: 1,698 (56.7%) wards with ANC TPR, 1,897 (64%) with mRDT TPR and 1,934 (65.3%) wards with incidence. For the remaining wards with data from only 1 HF, the average across all the estimated standard errors per indicator was used to represent the uncertainty of the corresponding indicator (Fig S5).


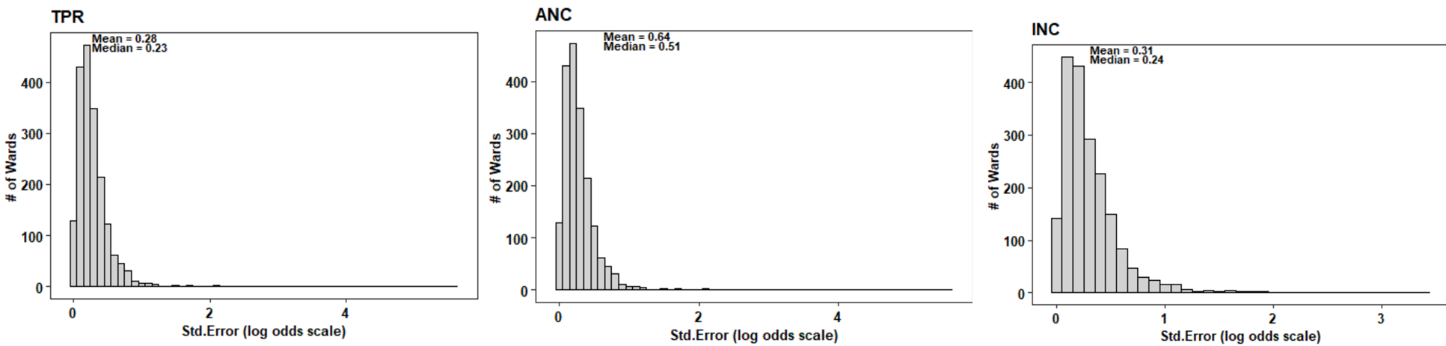


**Fig S5:** Distribution of the estimated standard errors for the wards with collected data from more than one health facility.

**Quantification of risk strata uncertainty: the probability of a ward being assigned to a risk stratum:**

For each ward, 1000 different sampled sets of indicator values were defined which were then separately used for running the micro-stratification procedure. Each set contained values for the three indicators. For each indicator, these values were sampled from a normal distribution with mean equal to the aggregated maximum indicator value per ward and standard deviation equal to the standard error estimated from the corresponding regression model on the log odds scale. Next, the micro-stratification procedure was separately conducted for each of the 1000 sampled sets of indicator values. Finally, for each ward, the proportion of times the micro-stratification yielded each risk category was computed and used to define the probability of a ward to be assigned to a risk stratum. The risk category with the highest assignment probability was selected to assign a ward to its corresponding risk stratum. The results were summarized through charts and maps and subsequently compared to the risk stratum obtained from the pragmatic approach.

**Figure S6:** Distribution of the routine indicators across wards by council prevalence

| **Council School Prevalence** | **Distribution in mRDT TPR** | **Distribution in API** | **Distribution in ANC TPR** |
| --- | --- | --- | --- |
| Very Low  (*Pf*PR_5-16_ <1%) | 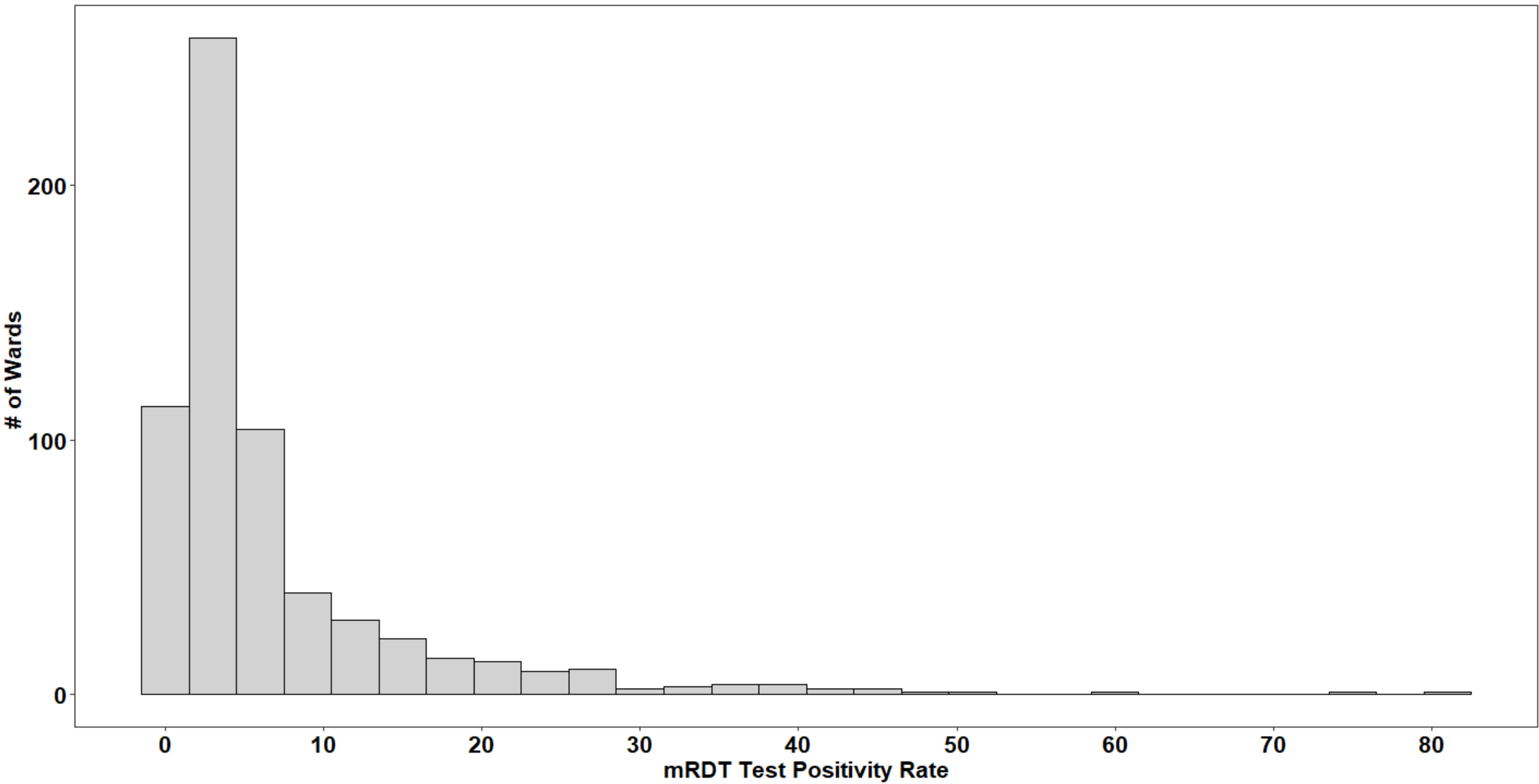 | 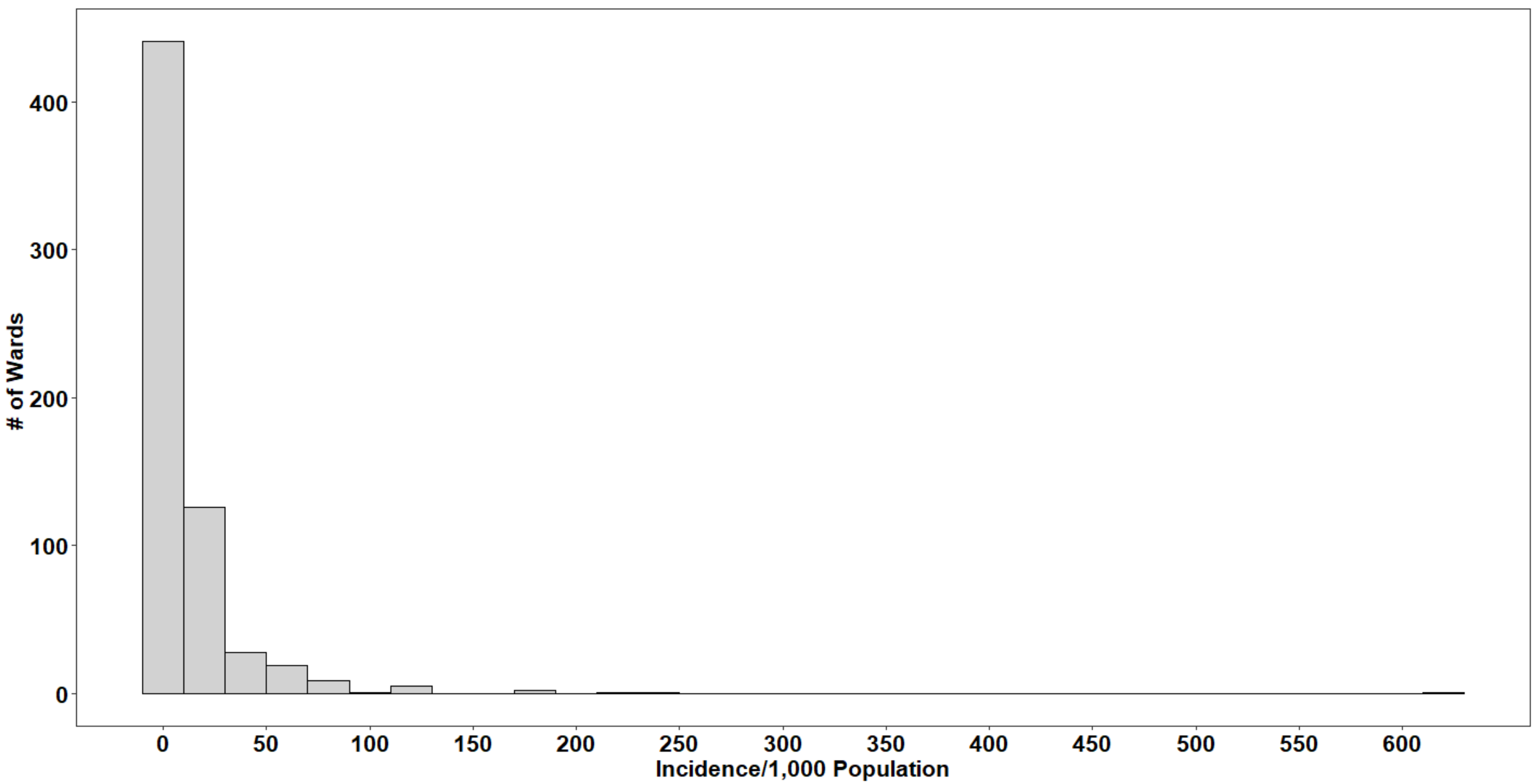 | 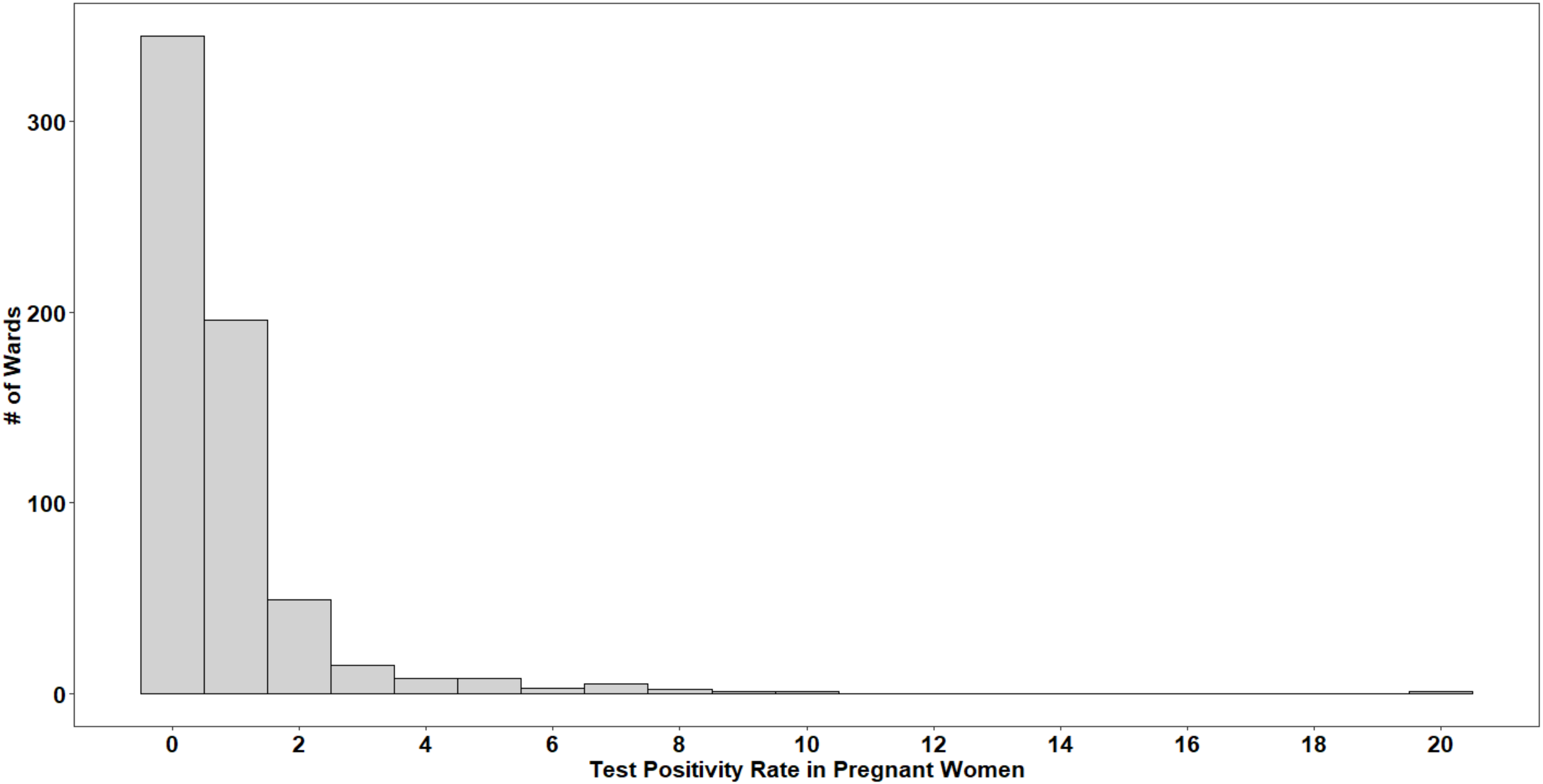 |
| Low  (*Pf*PR_5-16_ 1-5%) | 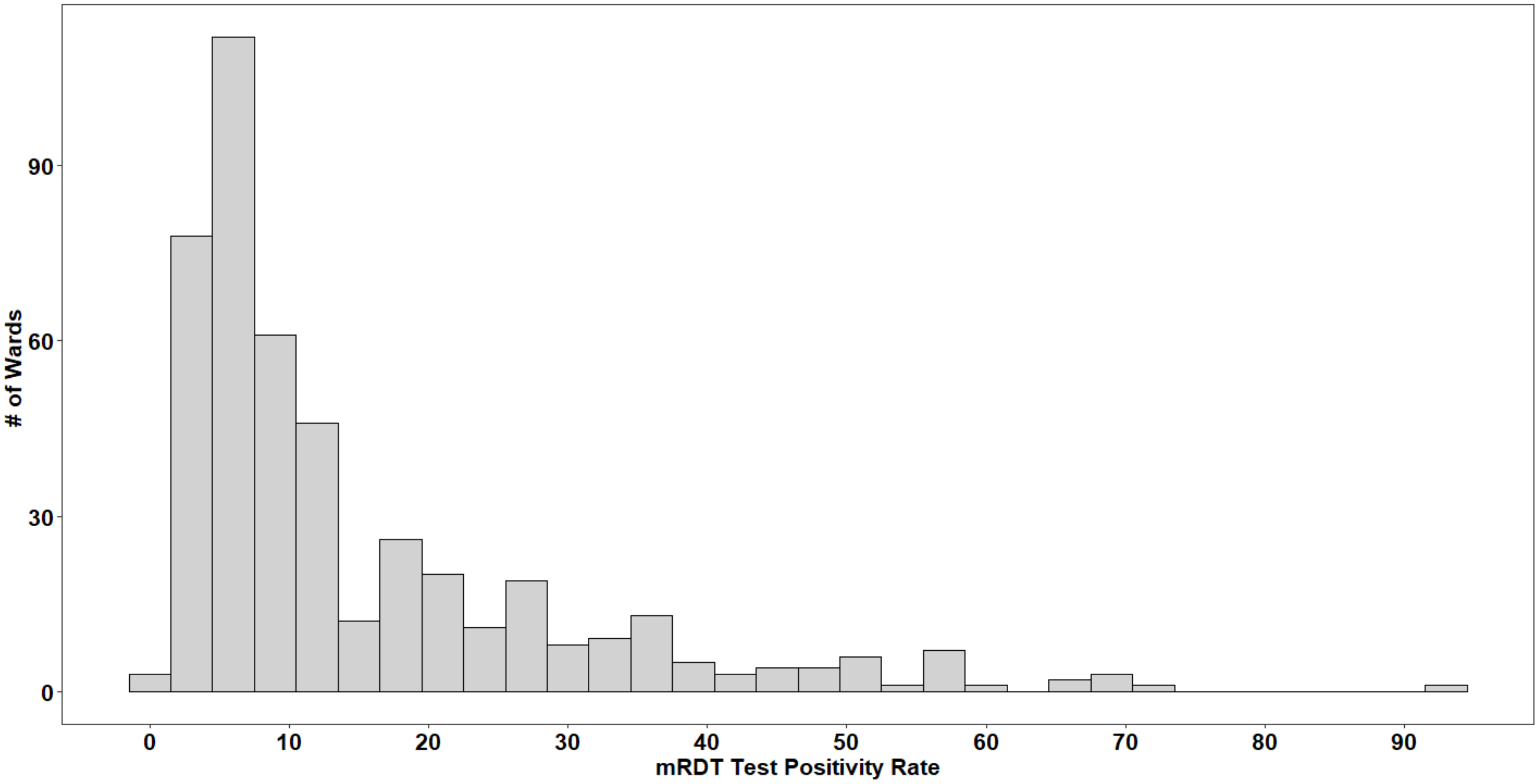 | 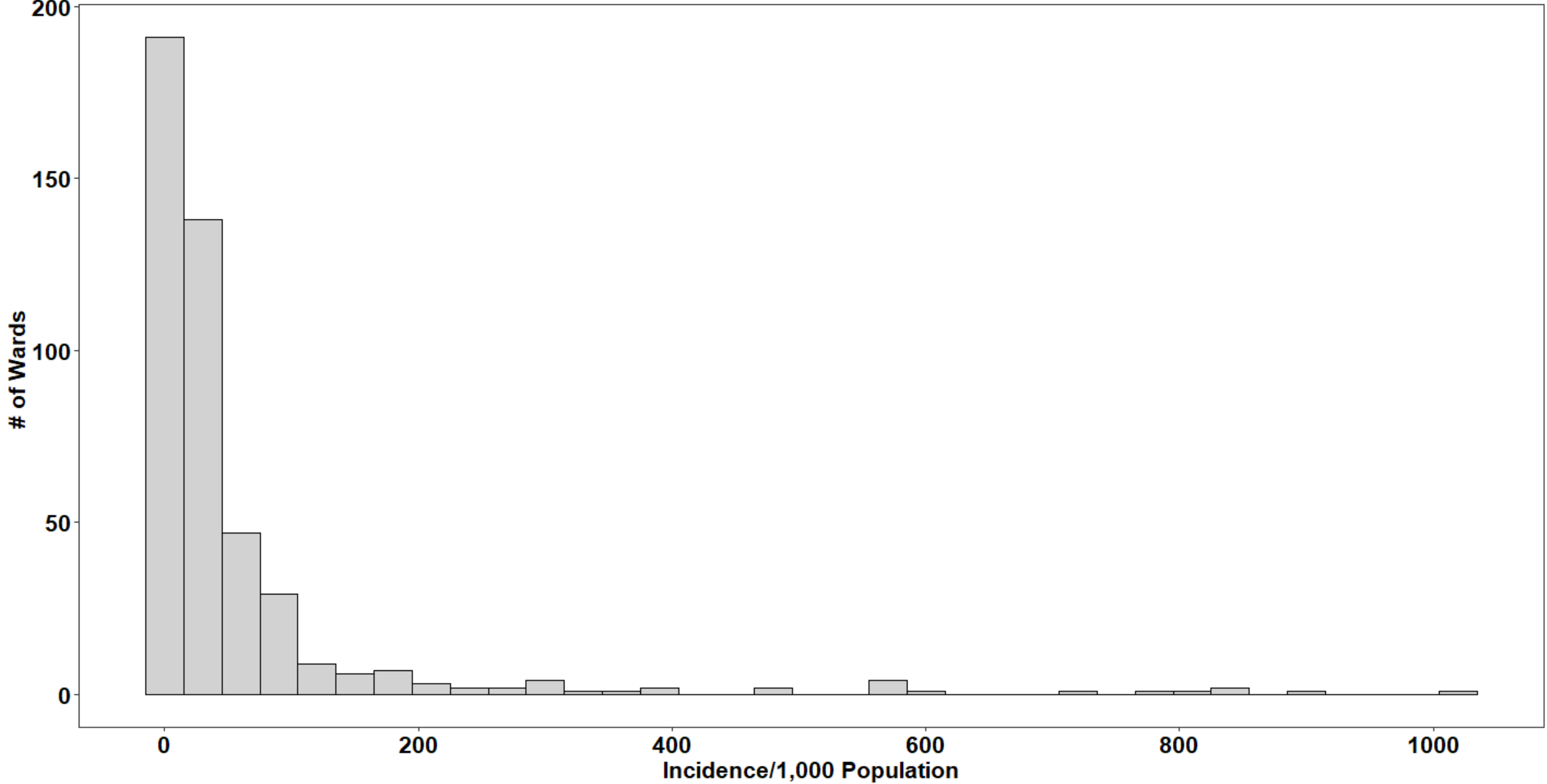 | 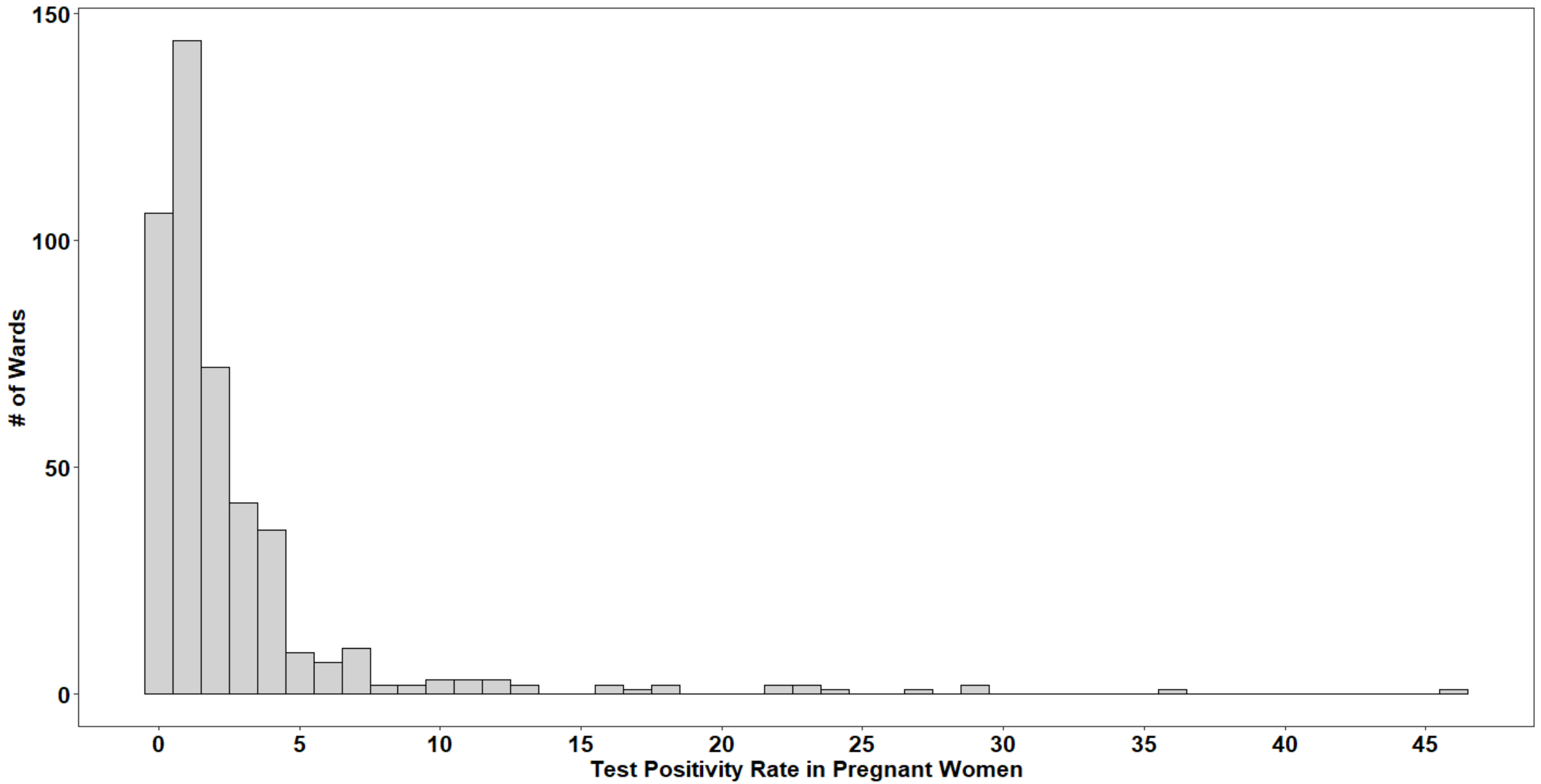 |
| Moderate  (*Pf*PR_5-16_ 5-30%) | 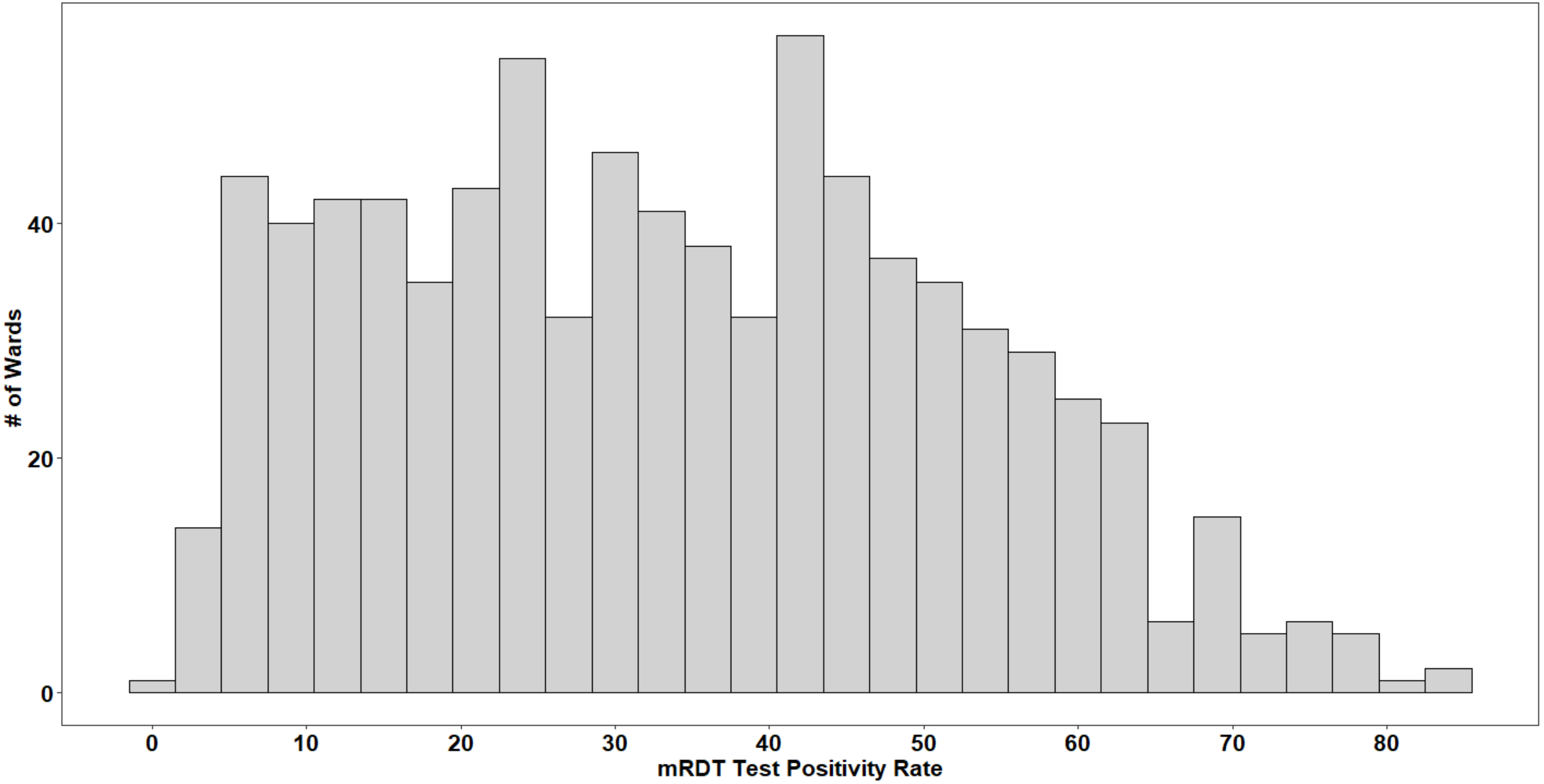 | 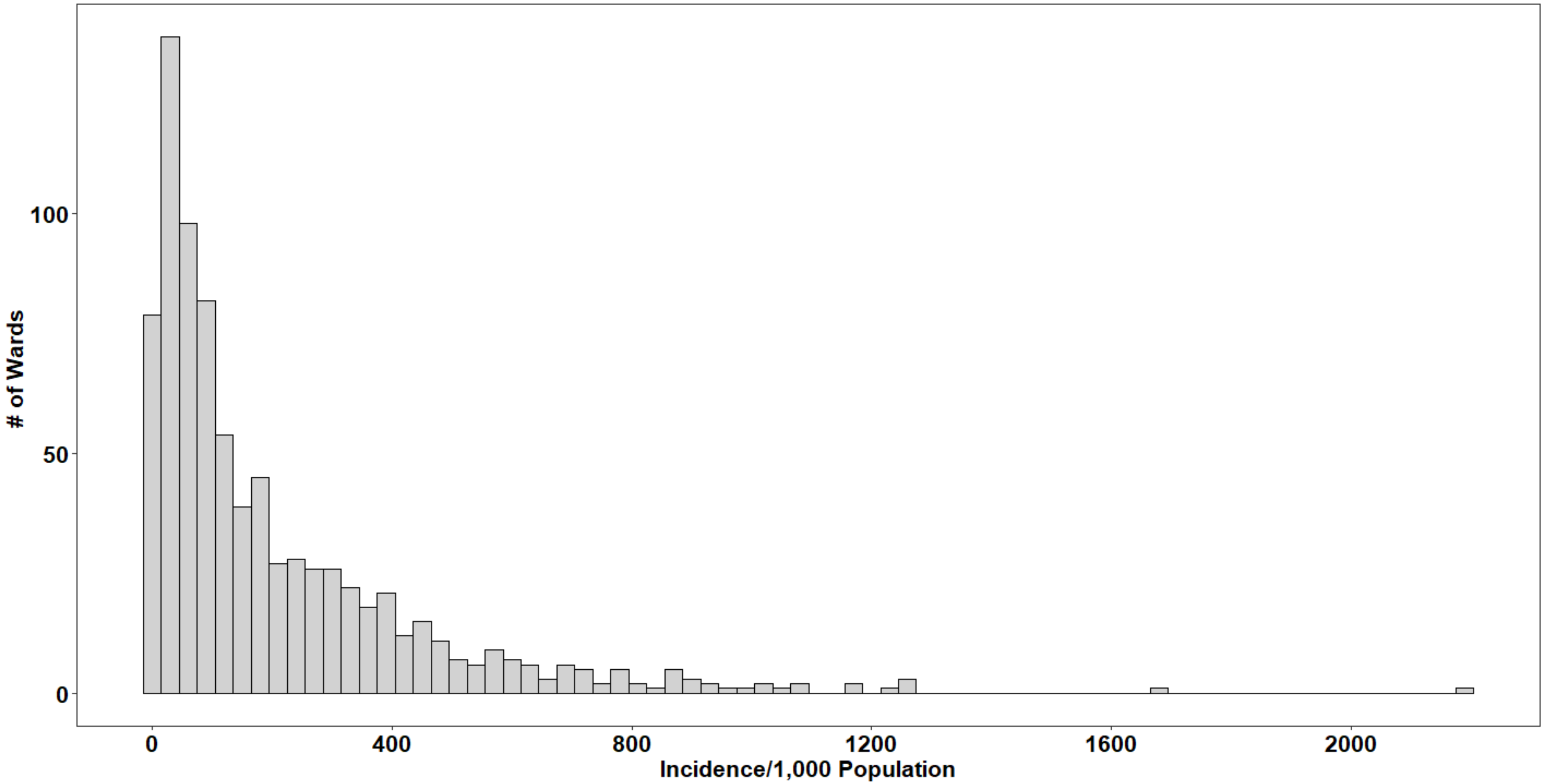 | 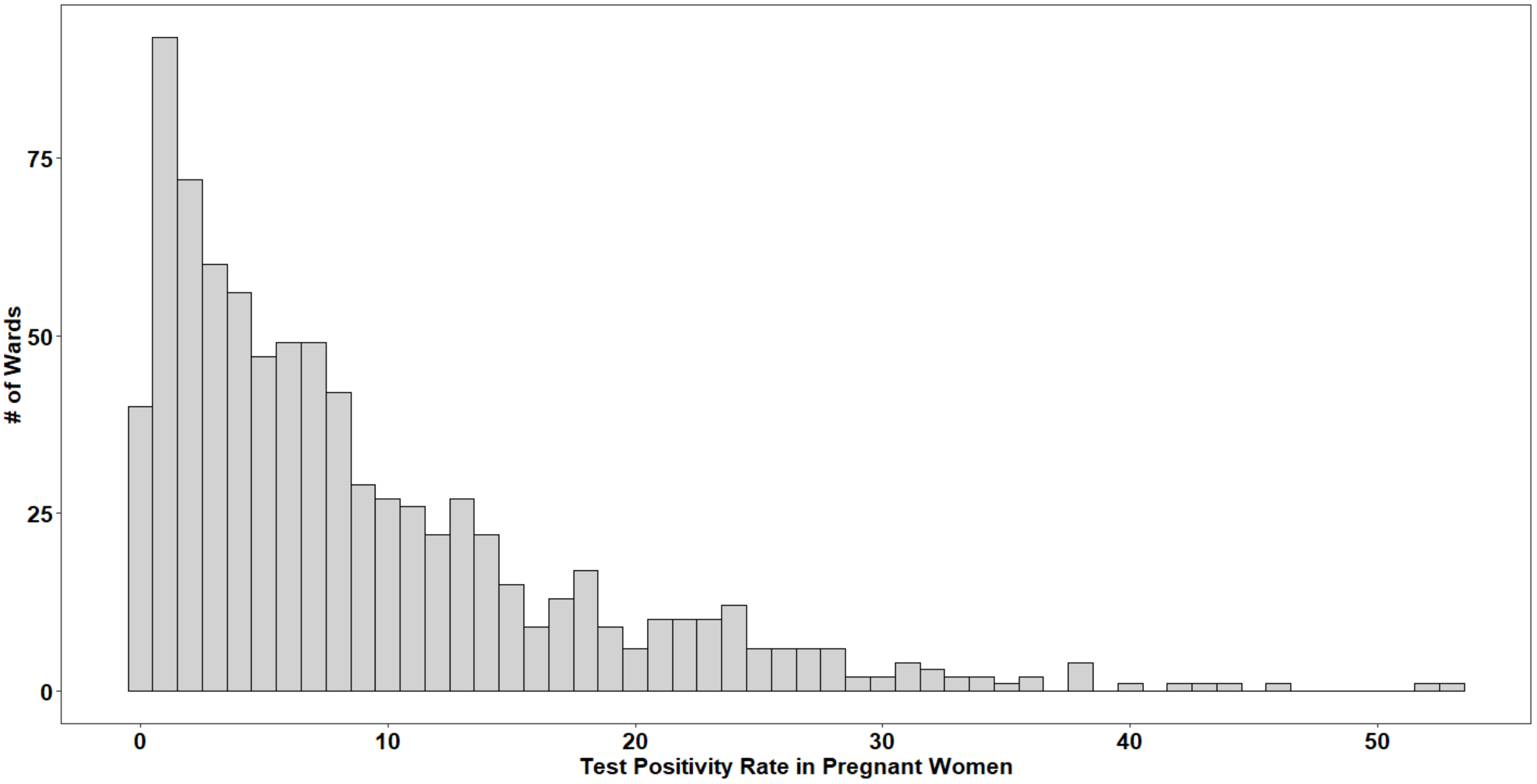 |
| High  *Pf*PR_5-16_ >30% | 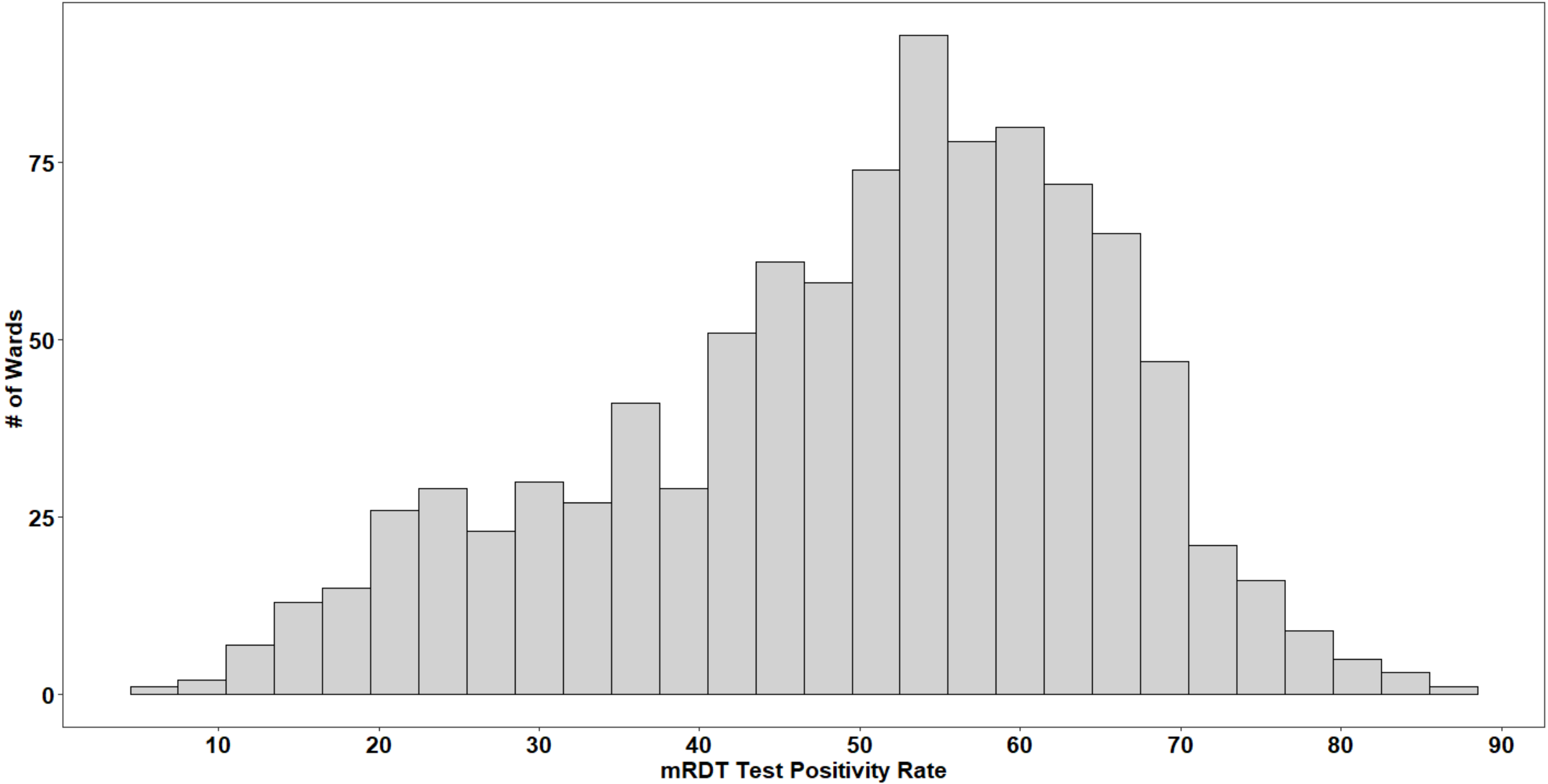 | 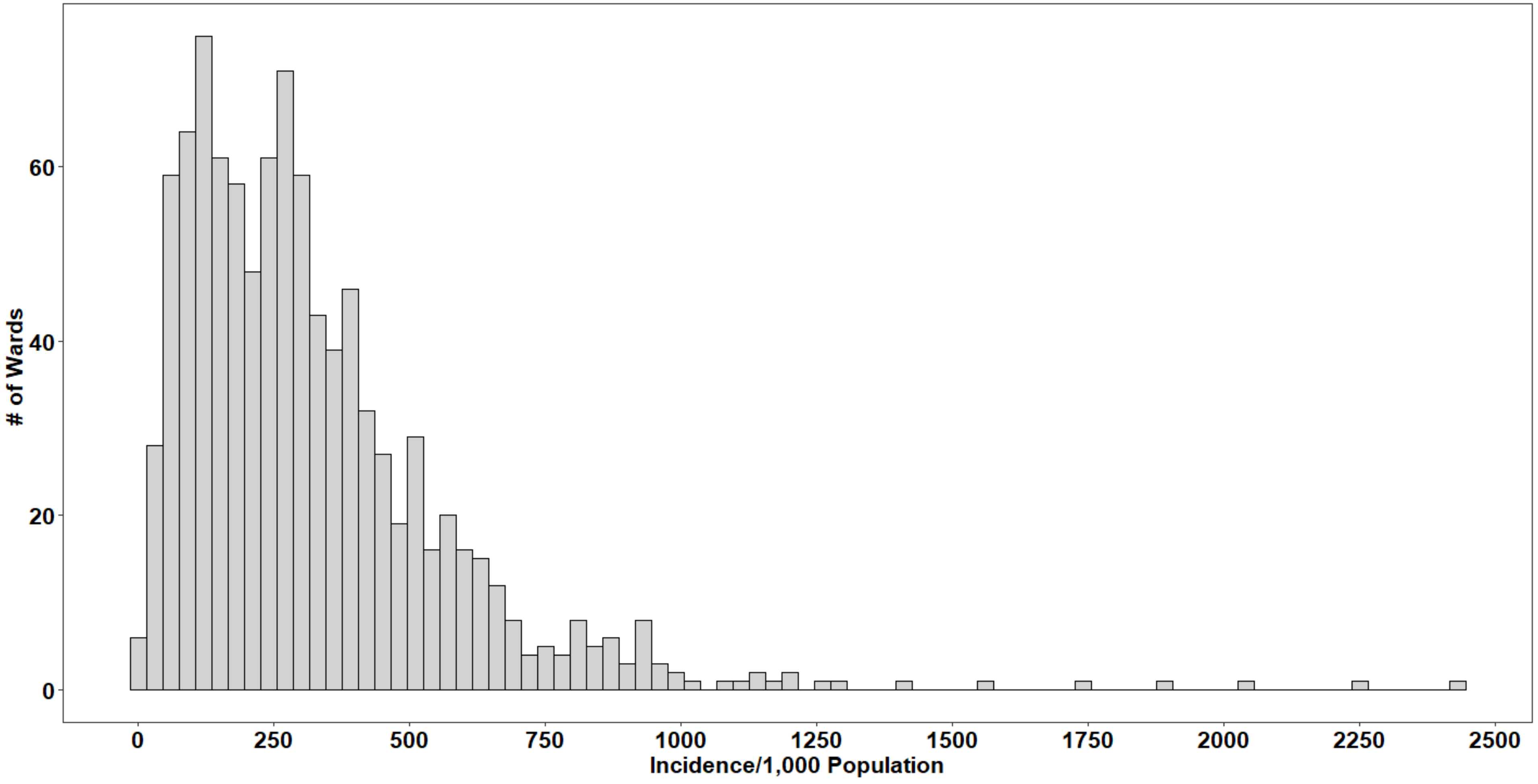 | 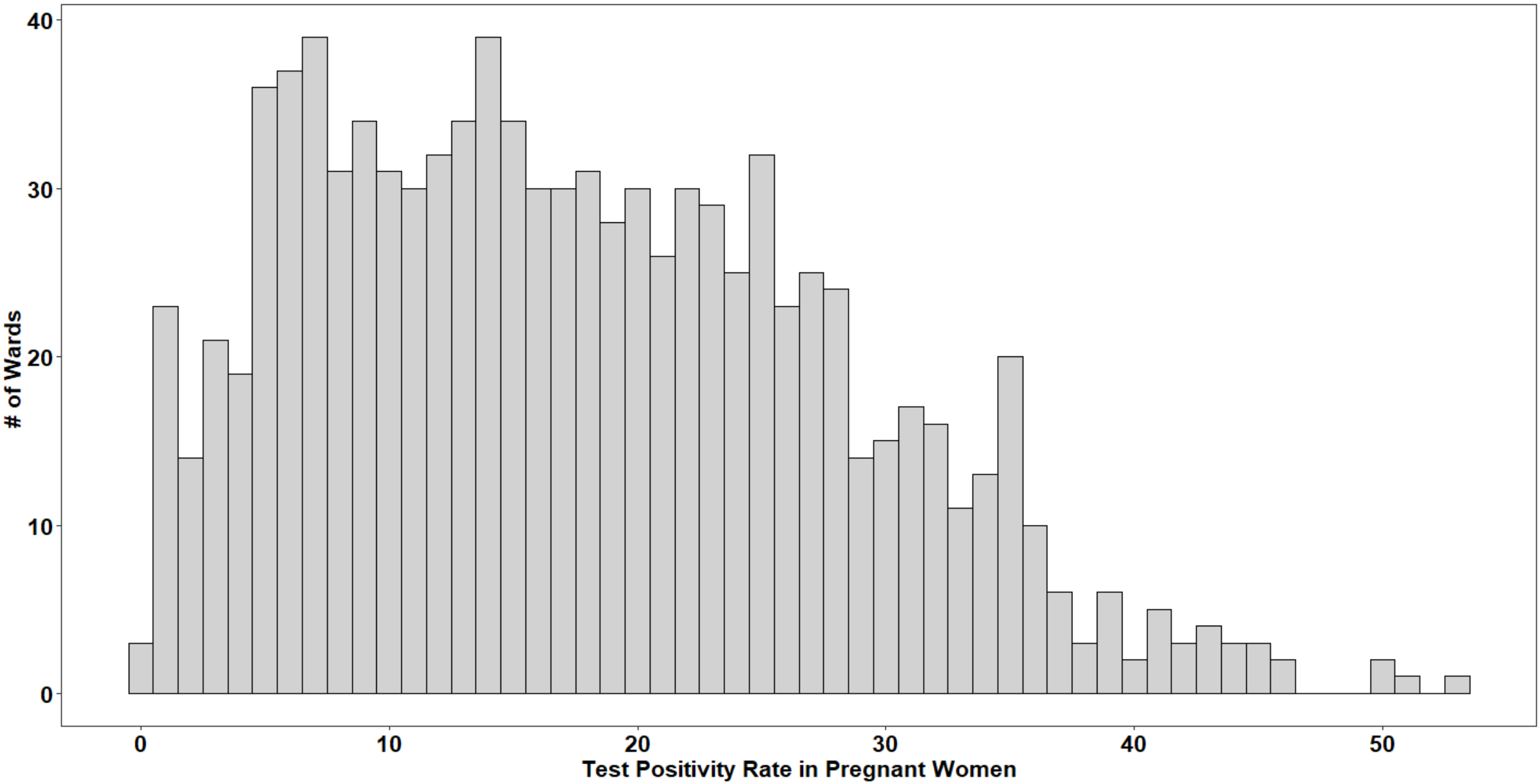 |

**Figure S7**: Spatial distribution at ward level of the maximum values of the mean annual malaria risk by type of indicator

| **mRDT TPR** | **API per 1,000 Population** | **ANC TPR** |
| --- | --- | --- |
| 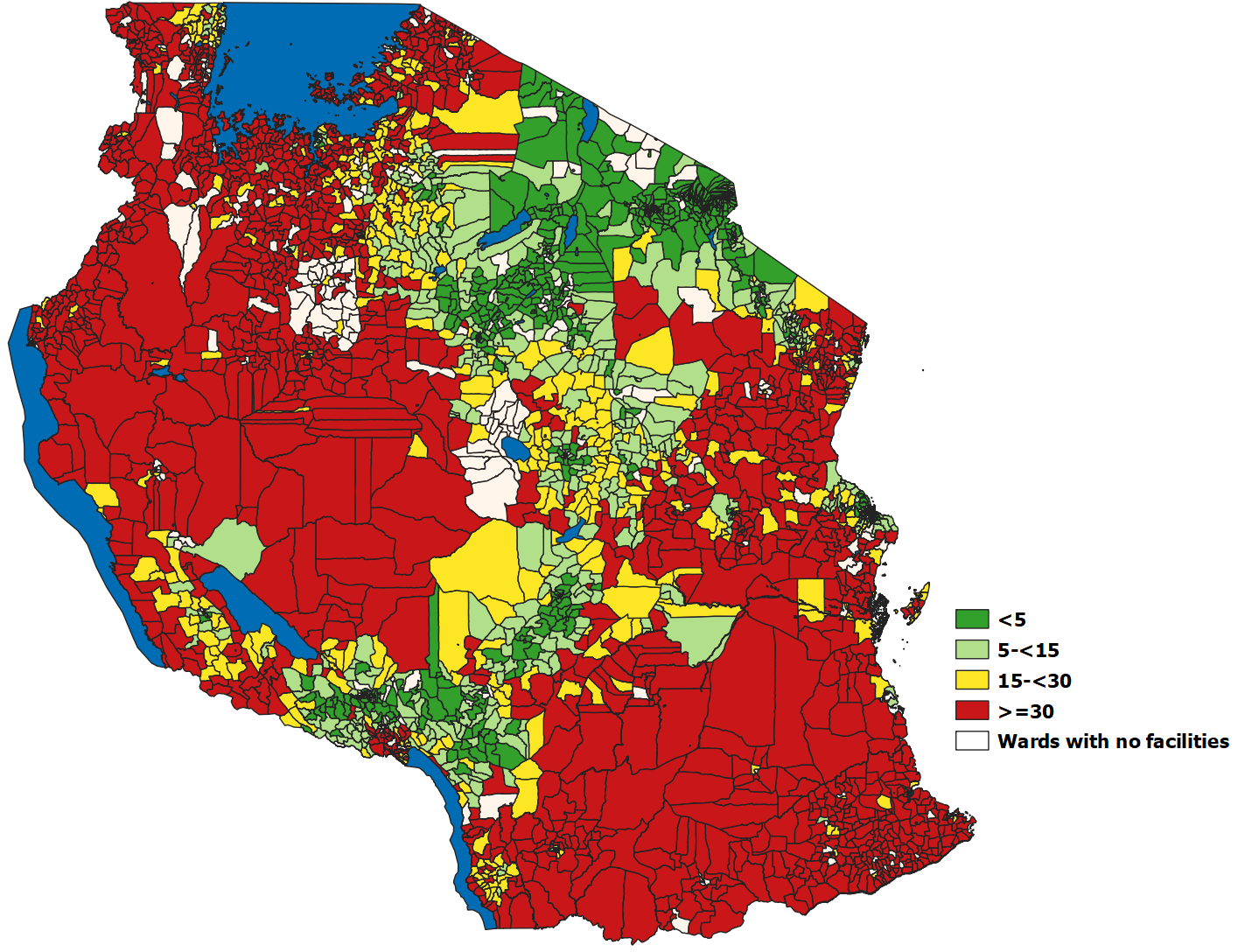 | 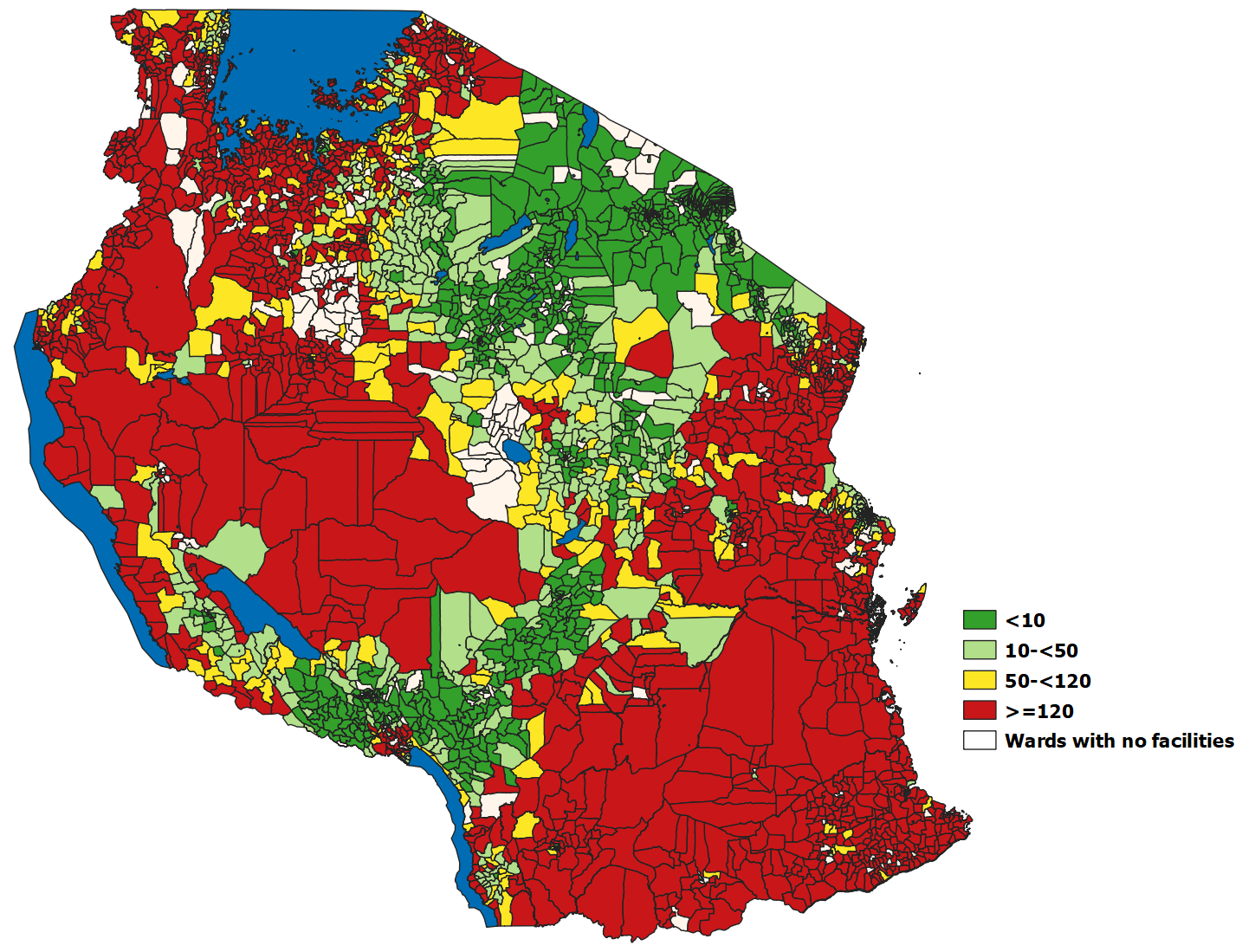 | 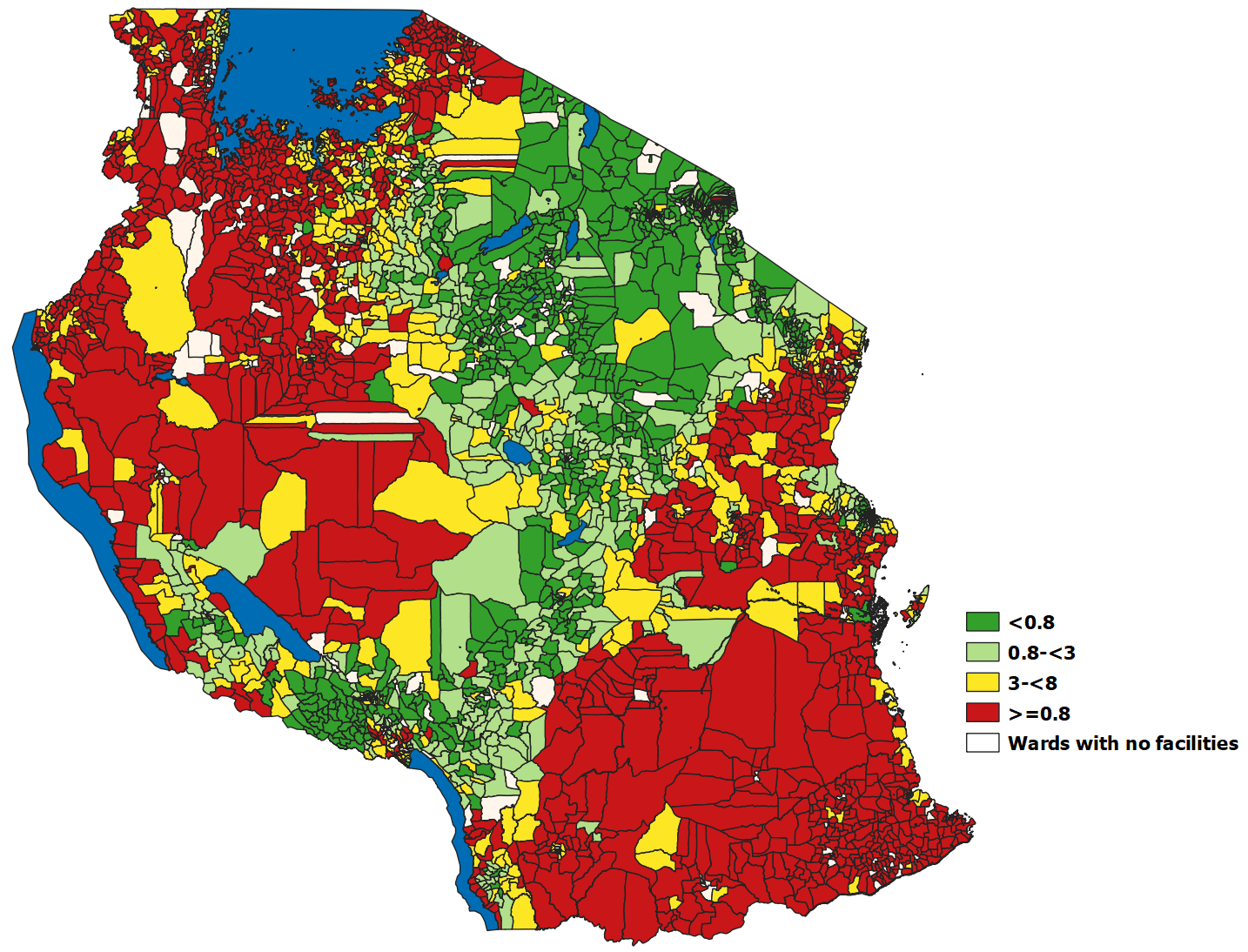 |

**Table S3** in the excel sheet shows the proportion of heterogeneity per council.

Laboratory ANC


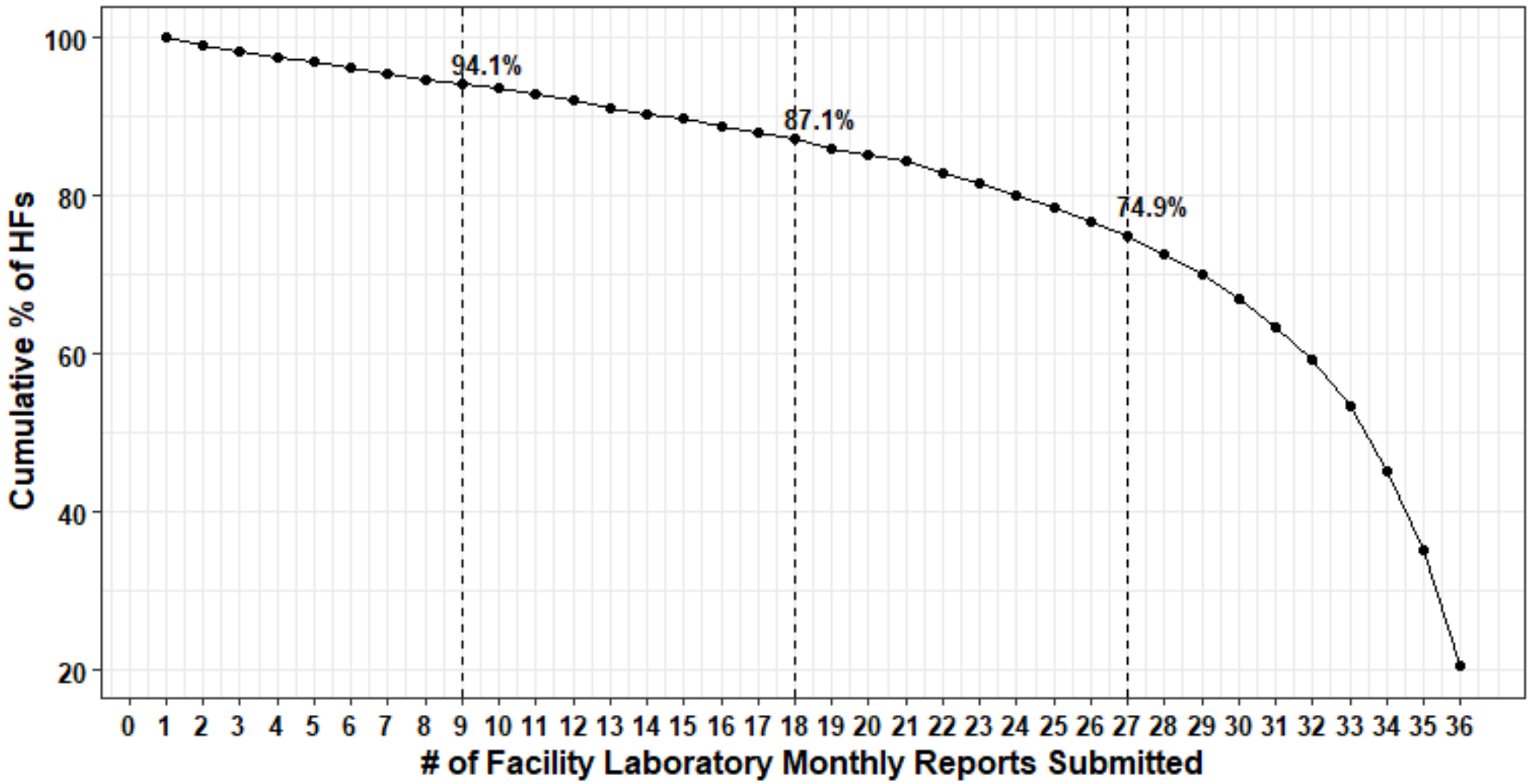

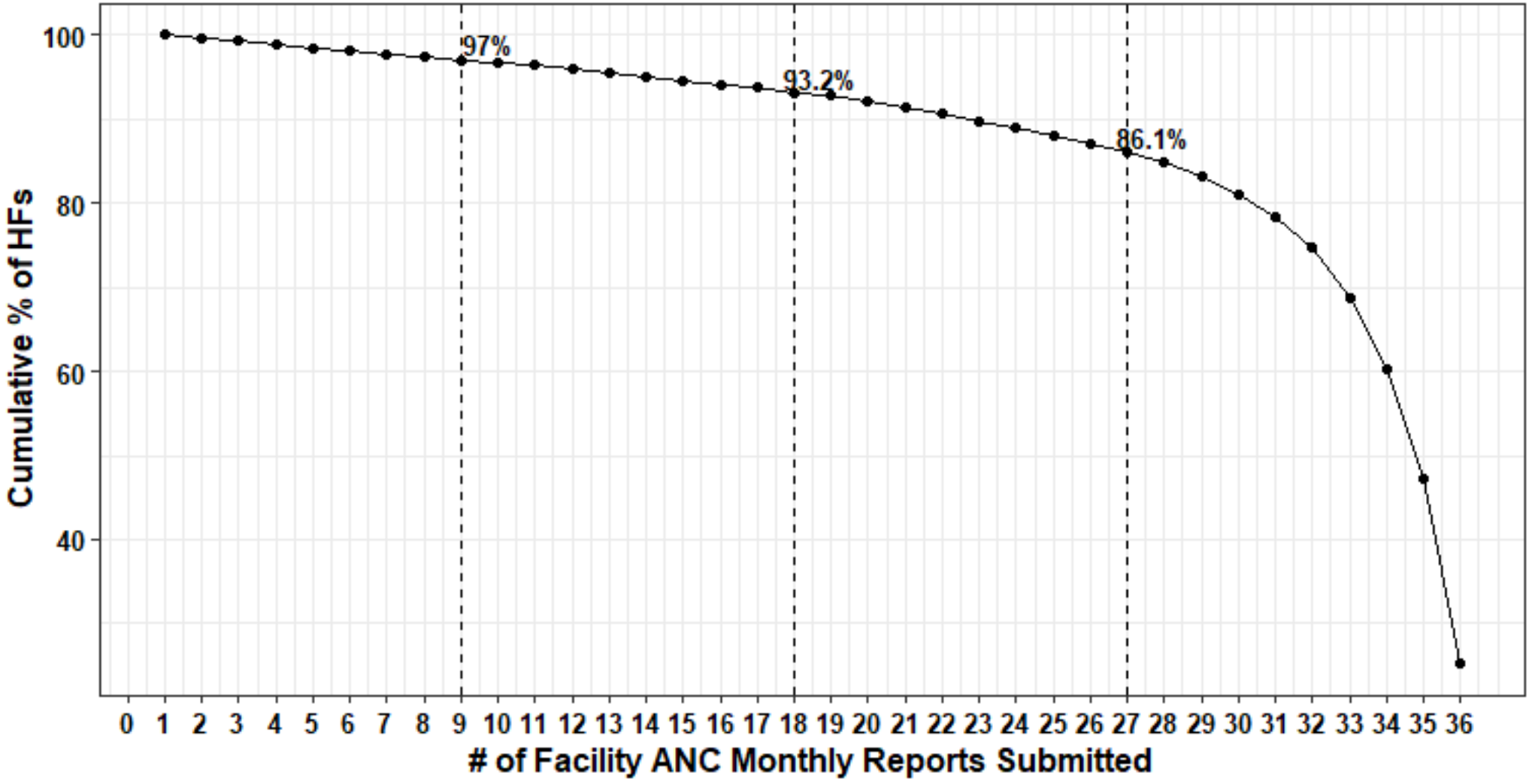


**Figure S8:** Cumulative proportion of facilities submitting 0-36 monthly reports in the period 2017-2019 for laboratory and ANC reports in mainland Tanzania

**Figure S9:** Transmission risk across the wards of Bumbuli District Council


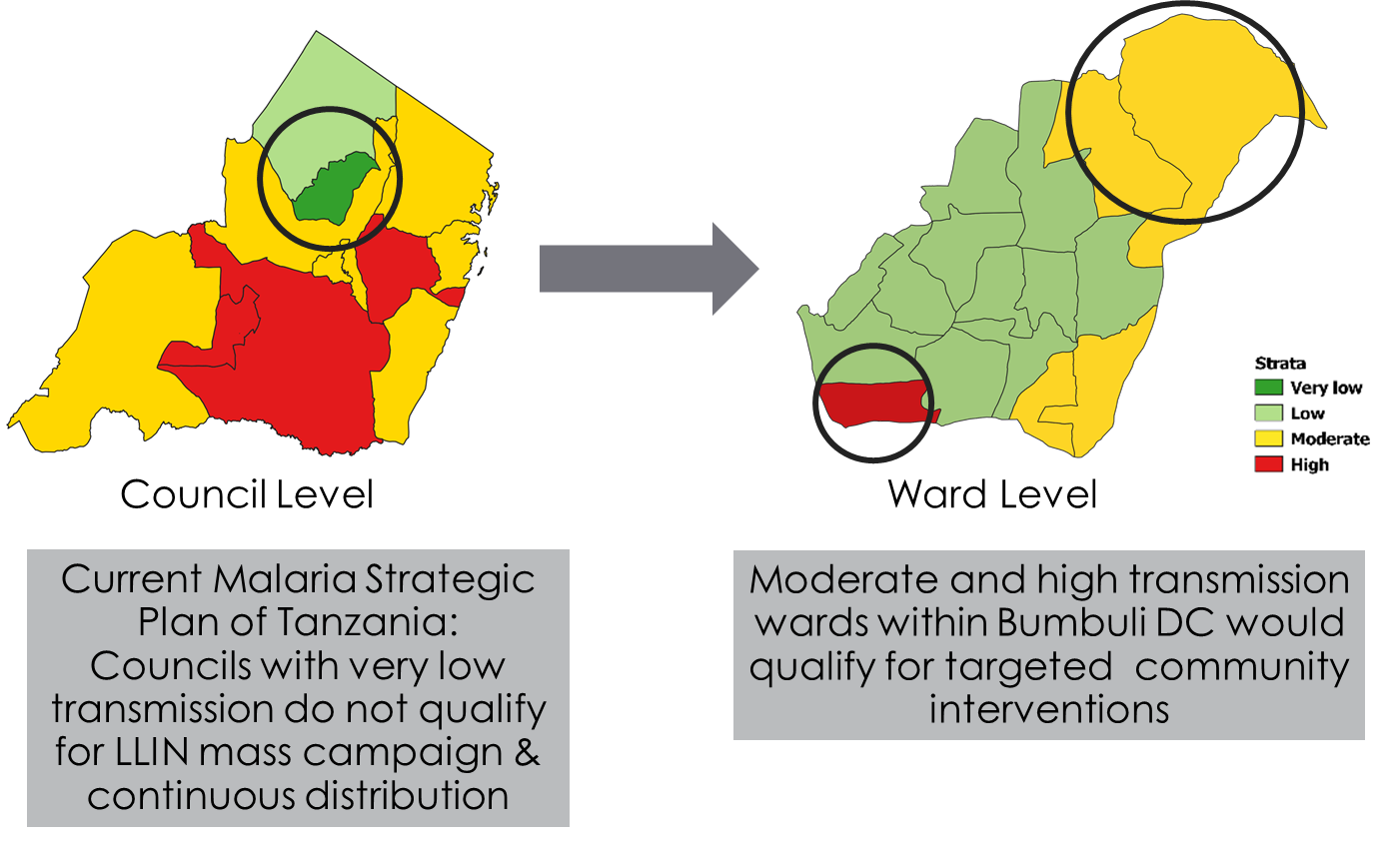


| **ANC TPR** | **TPR** | **API** |
| --- | --- | --- |
| **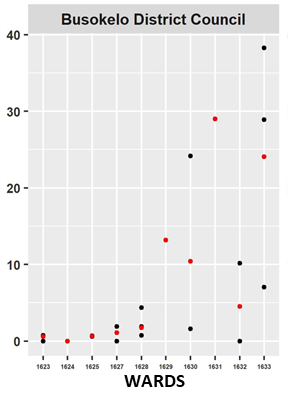** | **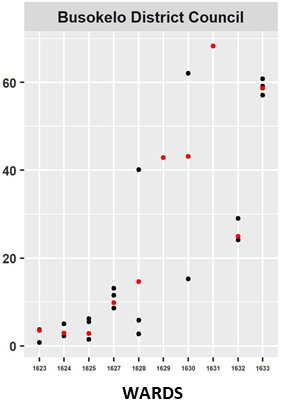** | **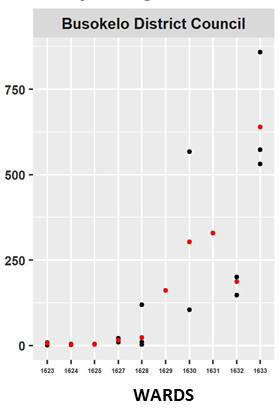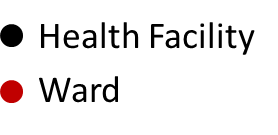** |

**Figure S10:** Distribution of the routine indicators across health facilities within the wards of Busokelo DC. The dots represent the values of the routine indicators at the health facility (black) and aggregated at the ward level (red).

**Text S4: The probability of each ward being assigned to a risk strata**

We used a sampling-based approach to estimate the uncertainty in the routine indicators by defining an assignment probability of a ward to a risk stratum. The wards assigned to the very low and high risk strata had on average a higher assignment probability (>90%) than those assigned to the low and moderate strata (average assignment probability below 80% for the low stratum and below 70% for the moderate stratum, Figure S11).


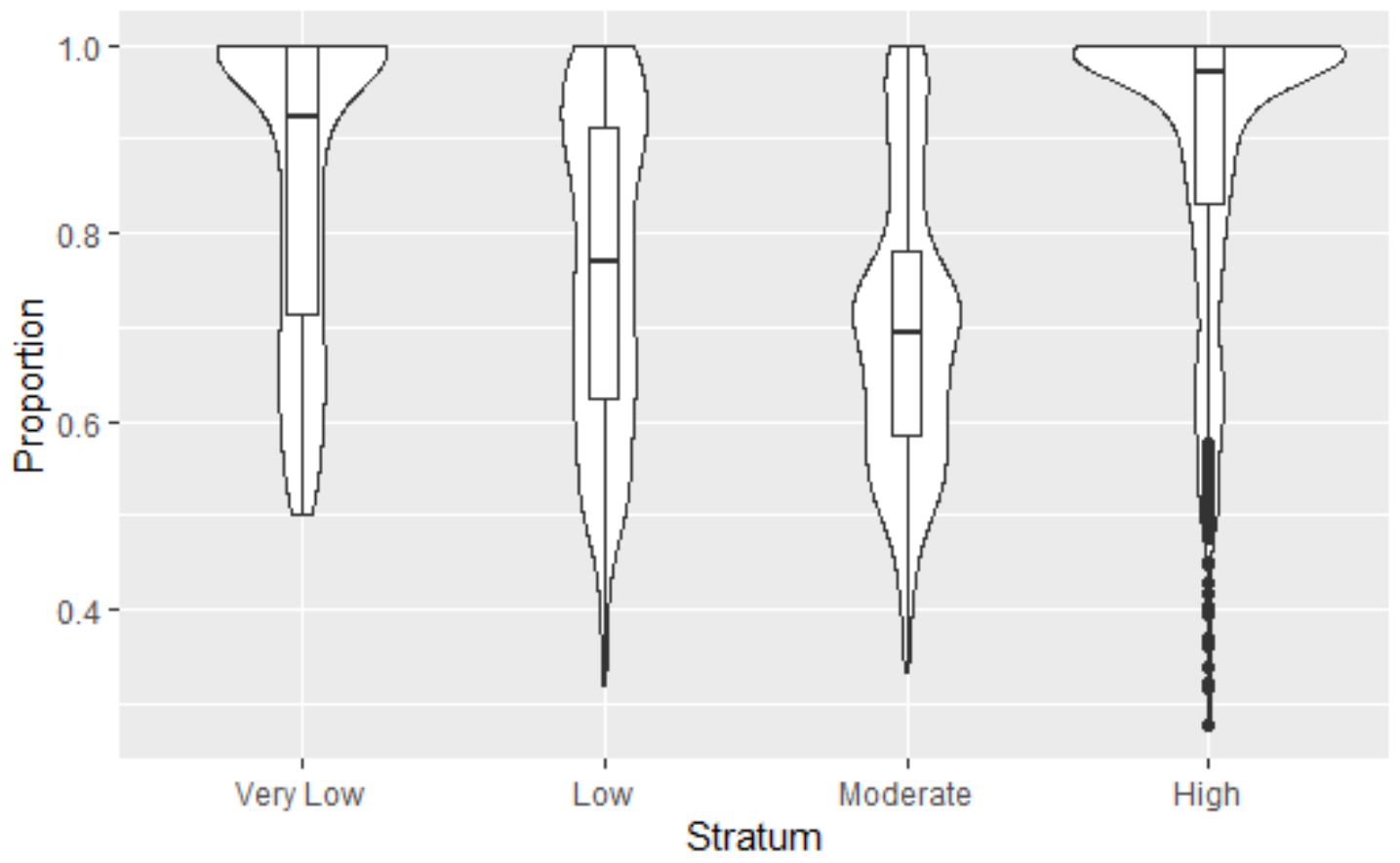


**Figure S11:** Distribution of the assignment probabilities of wards to malaria risk strata

Over 60% of the wards were assigned to the same risk stratum when the indicator variability was considered compared to the initial micro-stratification approach (Fig S12-S13).


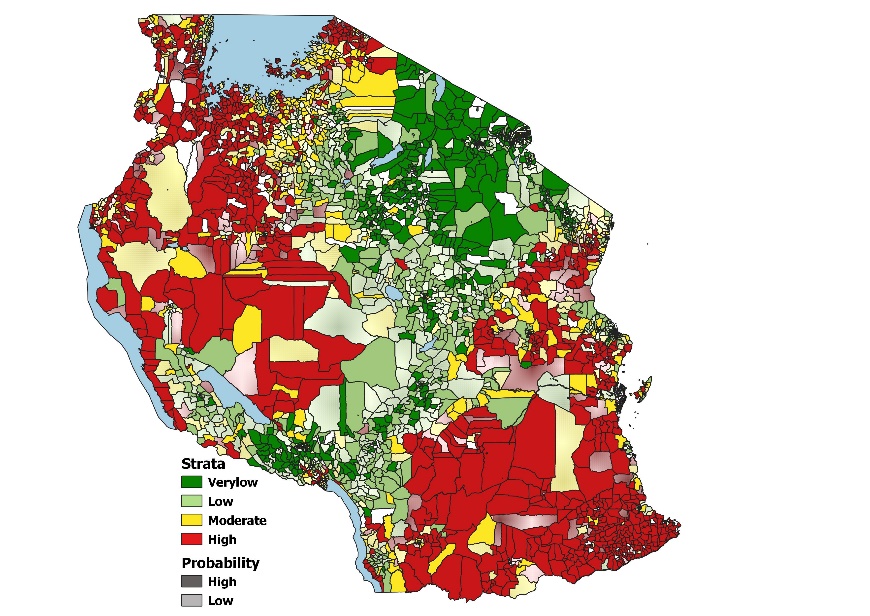

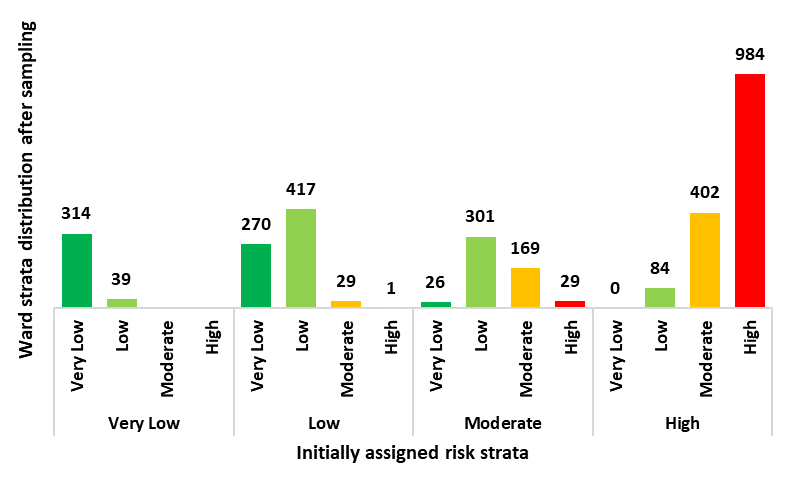


**Fig S12:** Micro-stratification risk map after accounting for indicator variability (the different color shades reflect the assignment probability)

The majority of wards (n=2129, 70%) were assigned to the risk stratum with probabilities larger than 70%, while the remaining 30% of the wards displayed variation in the indicators and had lower assignment probabilities (Figure S13). Out of the wards with assignment probability larger than 70%, 68% were assigned to the same strata as following the initial micro-stratification approach. The remaining 32% were mainly assigned to the immediate lower stratum compared to the initial approach. This was expected, as the initial micro-stratification approach is based on the maximum observed value for the routine indicators and is thus more conservative, avoiding to allocate wards to lower strata. For instance, after sampling and considering the standard errors of indicators, 38% of the wards initially assigned to the low stratum were allocated to the very low stratum, 57% of the wards initially assigned to the moderate strata were assigned to the low stratum and 27% of the wards initially assigned to the high were allocated to the moderate stratum.

| 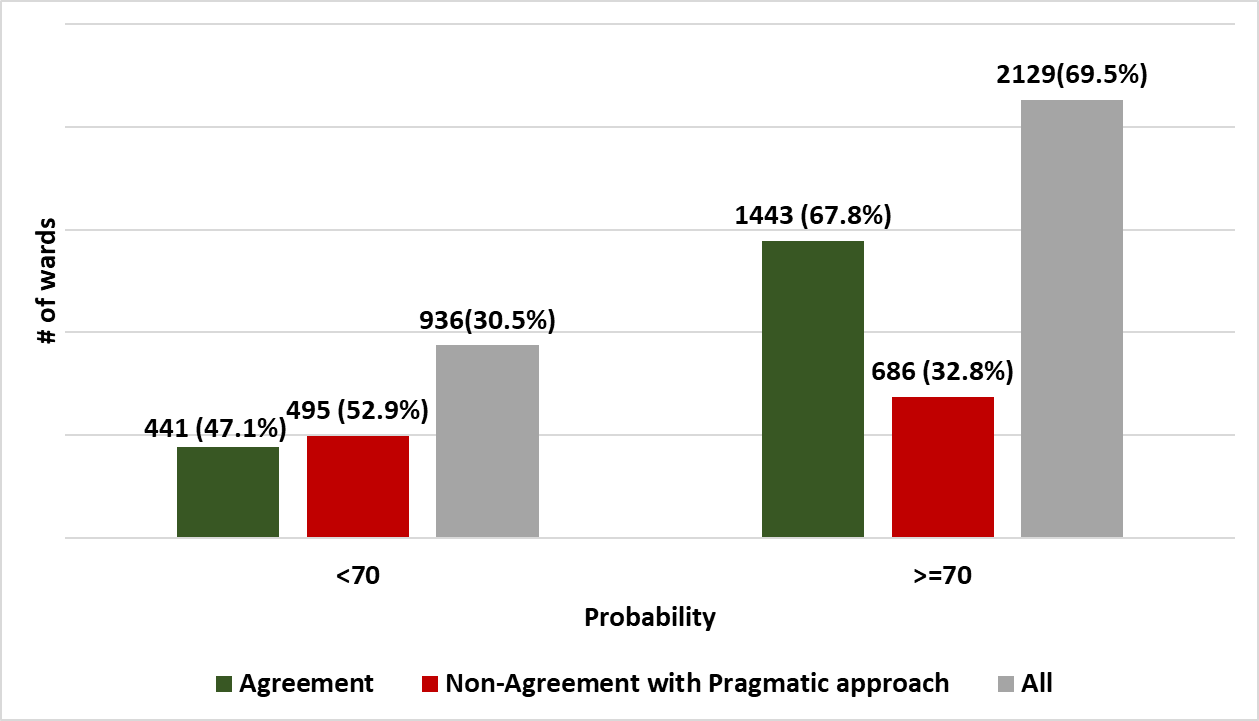 | |
| --- | --- |
| **Wards that Agree** | **Wards that don’t agree** |
| 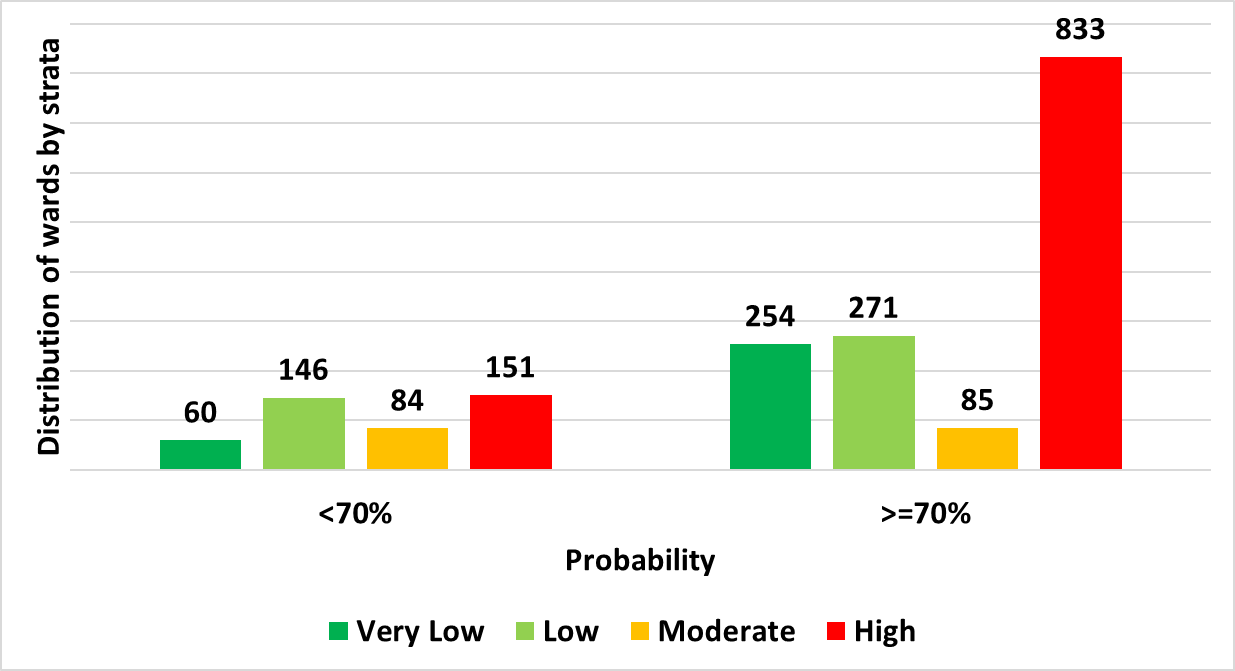 | 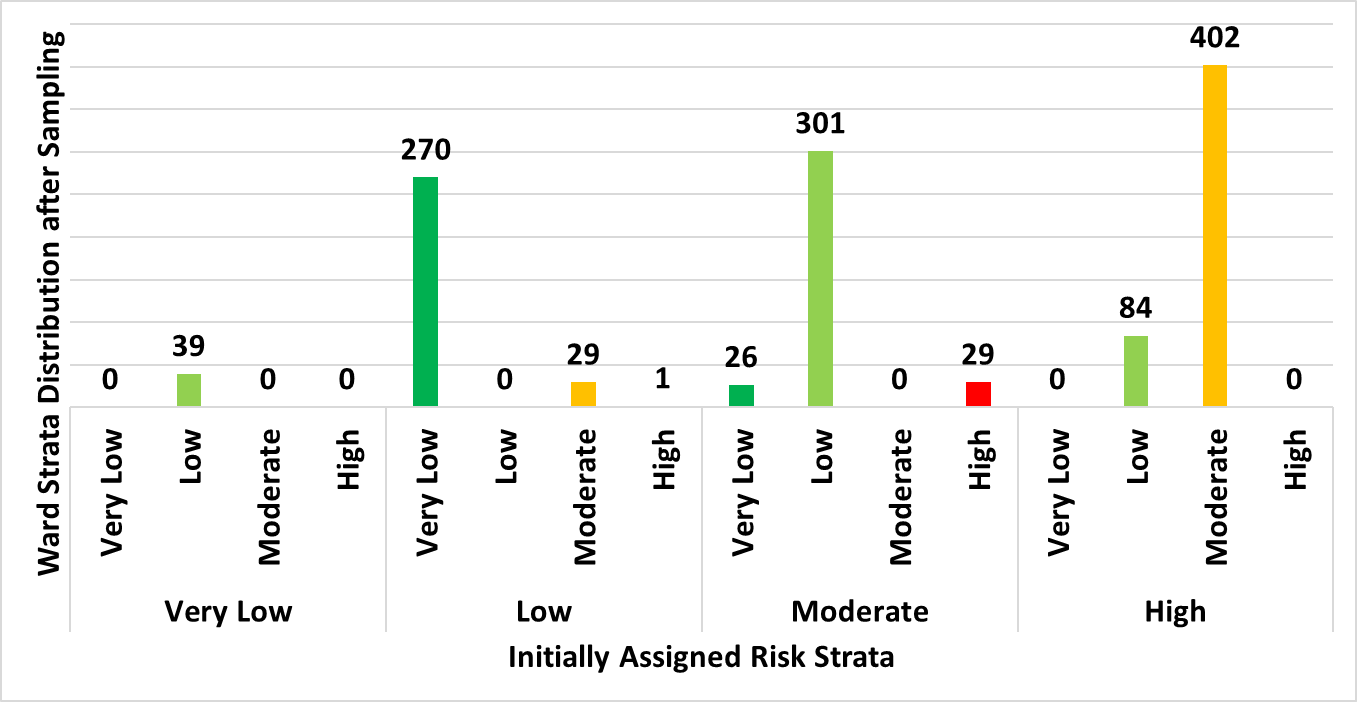 |

**Figure S13:** Comparison of the initially assigned risk strata vs after sampling disaggregated by probability
